# The critical role of *Toxoplasma gondii* GRA1 in nutrient salvage

**DOI:** 10.1128/mbio.01242-25

**Published:** 2025-06-27

**Authors:** Bolin Fan, Biyun Qin, Mingjun Li, Yuchao Zhu, Fuqiang Fan, Hao Xu, Lilan Xue, Yukun Chen, Ming Pan, Bang Shen

**Affiliations:** 1 State Key Laboratory of Agricultural Microbiology, College of Veterinary Medicine, Huazhong Agricultural University 47895, Wuhan, Hubei, China; 2 Hubei Hongshan Laboratory, Wuhan, Hubei, China; 3 Shenzhen Institute of Nutrition and Health, Huazhong Agricultural University 47895, Wuhan, Guangdong Province, China; 4 Shenzhen Branch, Guangdong Laboratory for Lingnan Modern Agriculture, Genome Analysis Laboratory of the Ministry of Agriculture, Agricultural Genomics Institute at Shenzhen, Chinese Academy of Agricultural Sciences 441191, Shenzhen, Guangdong Province, China; University of Wisconsin-Madison, Madison, Wisconsin, USA

**Keywords:** dense granule protein, intravacuolar network, GRA17, pantothenate, TCA cycle, mitochondrion

## Abstract

*Toxoplasma gondii* is an obligate intracellular pathogen that infects humans and many animals. It harbors unique secretory organelles to facilitate its parasitic lifestyle, including dense granules that secrete diverse proteins to different destinations. Yet the biological functions of these secretory proteins are mostly unknown. Here, we examined the roles of GRA1, the first dense granule protein discovered. GRA1 is secreted to the parasitophorous vacuole (PV), and its conditional depletion led to growth arrest of tachyzoites, suggesting a crucial role of GRA1 for parasite propagation during acute infection. Furthermore, GRA1 inactivation resulted in severe metabolic defects, including reduced glycolysis and tricarboxylic acid cycle activities and a drop in cellular ATP levels. GRA1 depletion also disrupted the integrity of the mitochondrion and apicoplasts. Mechanistically, disruption of GRA1 altered the structures of membranous tubules in the PV, called the intravacuolar network (IVN), which are thought to mediate the transport of molecules in and out of the parasites. Consistently, GRA1 inactivation impaired the secretion of various GRA proteins to the PV membrane (PVM) or host cells, including GRA17 that is involved in nutrient uptake from host cells. In agreement with the mislocalization of GRA proteins, mutants lacking GRA1 had a strong defect in absorbing nutrients like glucose and pantothenate from host cells. Consequently, the parasites displayed slower growth and a higher tendency to differentiate into bradyzoites. Together, these results suggest that GRA1 plays a vital role in maintaining the structural integrity of the IVN, thus contributing to the scavenging of host nutrients.

**IMPORTANCE:**

*Toxoplasma gondii* critically relies on host nutrients for growth, but the underlying mechanisms are largely unknown. Here, we discovered that the secretory protein GRA1 in *Toxoplasma* played a crucial role in scavenging host nutrients to support parasite proliferation. GRA1 is the first reported GRA protein, but its function remains enigmatic since its discovery in 1989. GRA1 is secreted to the PV from the dense granules, which harbor many proteins that traffic to PV, PVM, and even host cells to perform diverse functions, including forming channels on the PVM to take up host nutrients to establish parasitism. GRA1 disruption impaired the structure of IVN and abolished the targeting of other GRA proteins to their destinations, which compromised the parasite’s ability to import nutrients from the hosts. These findings reveal the functional mode of GRA1 in *T. gondii* and highlight its potential as a target for developing new interventions against toxoplasmosis.

## INTRODUCTION

*Toxoplasma gondii* is a widespread parasite belonging to the phylum Apicomplexa. It infects a broad range of warm-blooded animals and roughly one-third of the human population (1, 2). This parasite has a complex life cycle that contributes to its transmission. During the acute infection, tachyzoites proliferate rapidly and cause damage to host tissues, which are responsible for the symptoms of the disease (3, 4). As an opportunistic pathogen, *T. gondii* infection is typically asymptomatic in otherwise healthy individuals since acute infections are quickly controlled by the hosts’ immune responses. Consequently, the parasites switch to a semi-dormant stage called bradyzoites to form a chronic infection, when the parasites proliferate very slowly and are encysted to facilitate long-term persistence in infected hosts. However, *T. gondii* infections can be life-threatening in immune-compromised individuals, including AIDS patients and those receiving immunosuppressive therapies (5–8). Reactivation of existing chronic infections in such patients may also cause severe complications. But at this moment, there is no effective treatment for chronic toxoplasmosis.

*T. gondii* contains a number of secretory organelles that are unique to apicomplexan parasites. These include micronemes, rhoptries, and dense granules, which secrete proteins to different cellular compartments and play diverse roles (9–12). Several micronemal proteins, including MIC2 and AMA1, and rhoptry proteins, including RON2, RON4, and RON8, are key for parasite invasion as they mediate the essential recognition between parasites and host cells, as well as the establishment of the moving junction, through which the parasite enters the host cell. Once inside the host cell, the parasite is enclosed in a PV, which is separated from the host cytosol by the PV membrane (PVM). The PVM is non-fusogenic with host lysosomes so that it protects the parasite from the host’s lysosomal degradation (13–18). However, the PVM is also a physical barrier between the parasite and the host cytoplasm, limiting the parasites’ interaction with the host cells and restricting direct access to host-derived nutrients (16). Consequently, *T. gondii* secretes a wide variety of proteins to the PVM and even host cells to manipulate host cell activities for its growth and survival (19, 20). These include GRA proteins like GRA17, GRA23, and GRA72 that localize to the PVM to form channels, allowing molecules smaller than 1.3 kDa to enter the PV from host cytoplasm (21–23).

As an obligate intracellular parasite, *Toxoplasma gondii* critically relies on host cells for nutrient supply (24–30). Although the parasite has the capacity to synthesize a variety of metabolites, primitive substrates like carbon sources for such metabolic activities are acquired from host cells. In addition, *Toxoplasma* is also auxotrophic for many metabolites that include purines, polyamines, cholesterol, and vitamin precursors like biotin and nicotinamide (31). The parasite manipulates host cell metabolic activities to satisfy its nutrient needs (32–36). In addition to forming channels on the PVM through dense granule proteins like GRA17, *T. gondii* employs additional GRA proteins, including Sec22b, GRA3, and MAF1, that localize to the PVM to recruit host organelles and facilitate nutrient acquisition from them (37–40). Furthermore, *T. gondii* exploits the host cell’s endosomal sorting complexes required for transport (ESCRT) and host organelle sequestration (HOST) pathways to internalize host cell nutrients and organelles (34, 35, 41). Once in the PV, nutrients like glucose, amino acids, and inorganic ions are further delivered to the parasite cytosol through various transporters in the plasma membrane of the parasites (24). Additionally, *T. gondii* also employs endocytic processes through the micropore to take up nutrients from the PV (42, 43).

The PV contains intravacuolar networks (IVN) formed by membranous tubules secreted by the parasites. The function of IVN has not been fully defined, but it was shown to be involved in nutrient exchange between the host cell and the PV (44, 45). A number of GRA proteins like GRA2 and GRA6 are localized to the PV and crucial for maintaining IVN integrity. Their deletions disrupted this network and impaired the scavenging of proteins and lipids from host cells (46–48). GRA1, the first reported GRA protein identified more than 35 years ago, is localized within both the IVN and the PV space (44, 49). Importantly, GRA1 is among the few GRAs that are crucial for parasite growth. However, its precise role remains unclear. In this study, we investigated the function of GRA1 in type I and type II strains of *T. gondii*. The results indicate that GRA1 is critical for the structural integrity of IVN, and its deletion led to altered trafficking of many GRA proteins, including GRA17, which is localized to PVM to mediate nutrient uptake into the PV. As a consequence, Δ*gra1* mutants had reduced salvage of nutrients from host cells and displayed severe growth defects.

## RESULTS

### GRA1 is essential for tachyzoite growth in the type I strain RH

**Fig 1:**
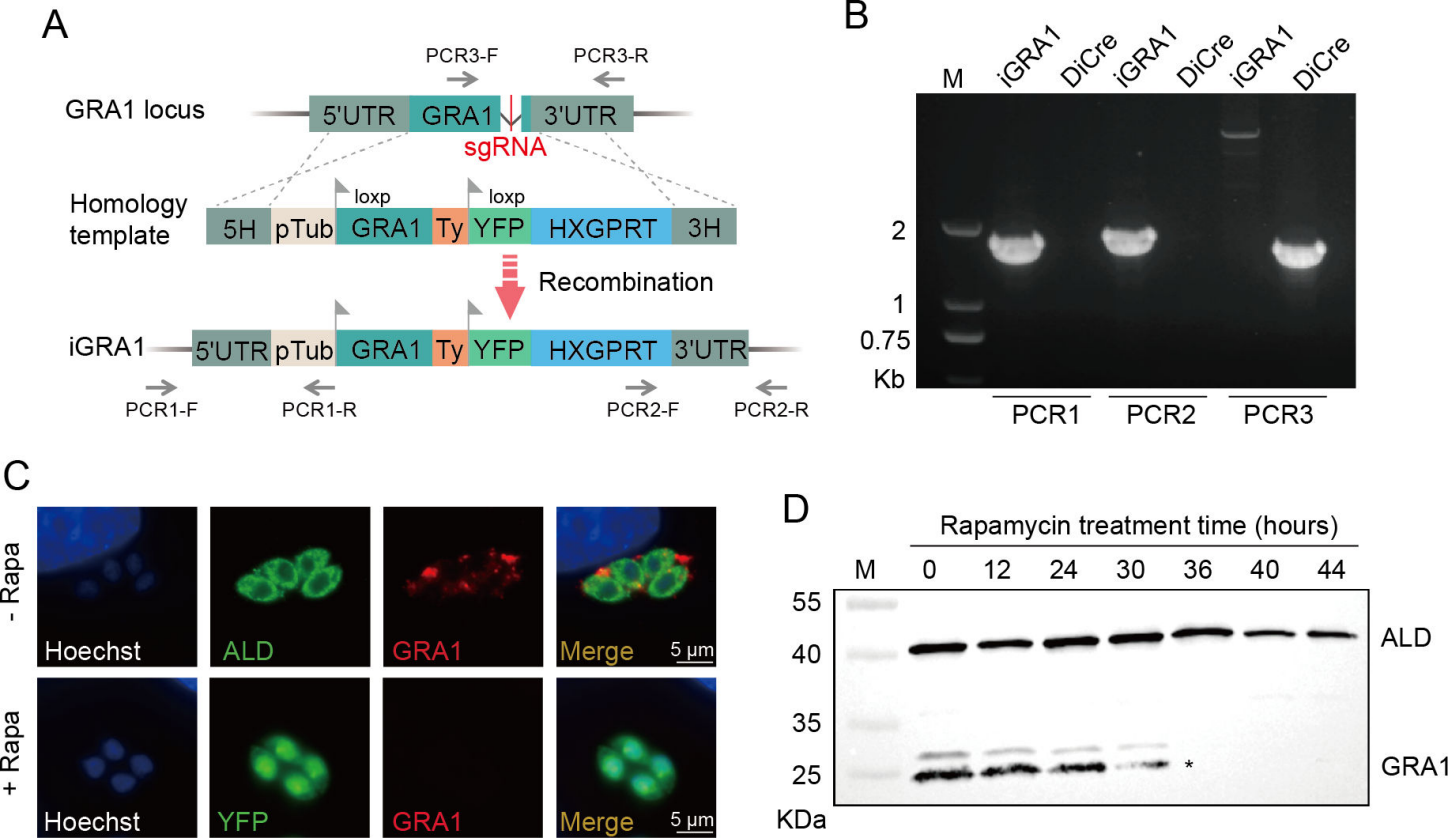
Construction of the GRA1 conditional knockout strain iGRA1. (**A**) Schematic illustration of floxing the GRA1 locus in the DiCre strain using CRISPR-/Cas9-assisted gene editing, to generate the iGRA1 strain. Primers used for diagnostic PCRs are indicated. (**B**) Diagnostic PCRs on the iGRA1 clone. (**C and D**) Conditional depletion of GRA1 in the iGRA1 strain, as determined by immunofluorescence assays (IFA) (**C**) or Western blotting (**D**), after treating parasites with 50 nM rapamycin. GRA1 was probed with a homemade mouse anti-GRA1 antibody, and the fructose-bisphosphate aldolase (ALD) was included as a reference. * denotes a cleavage product of GRA1.

To investigate the biological roles of GRA1 in tachyzoites, we constructed a conditional knockout strain RH-iGRA1 using the DiCre system (50), which allowed rapamycin-inducible deletion of GRA1. For this purpose, the endogenous GRA1 gene in the RH-DiCre strain was replaced with a floxed version of GRA1 (pTUB-loxp-TgGRA1-loxp-YFP), through CRISPR-/Cas9-assisted homologous recombination (Fig. 1A). The resulting iGRA1 strain was first examined by diagnostic PCR to confirm the floxing of GRA1 (Fig. 1B). Then, it was examined by immunofluorescent assays (IFA) to check the expression of GRA1 before and after rapamycin-induced GRA1 deletion. By design, treating the iGRA1 strain with rapamycin should induce the deletion of GRA1, meanwhile bringing YFP close to the pTub promoter so that its expression can be induced. Consistently, rapamycin treatment for 36 hours did induce YFP expression in the iGRA1 strain. In addition, those YFP^+^ vacuoles and parasites did not show GRA1 expression, suggesting GRA1 deletion in these parasites (Fig. 1C). Furthermore, Western blotting also showed that the GRA1 protein was reduced to undetectable levels in the iGRA1 strain after 36  h rapamycin treatment (Fig. 1D). These results suggest that GRA1 could be efficiently depleted by rapamycin treatment in the iGRA1 strain.

**Fig 2:**
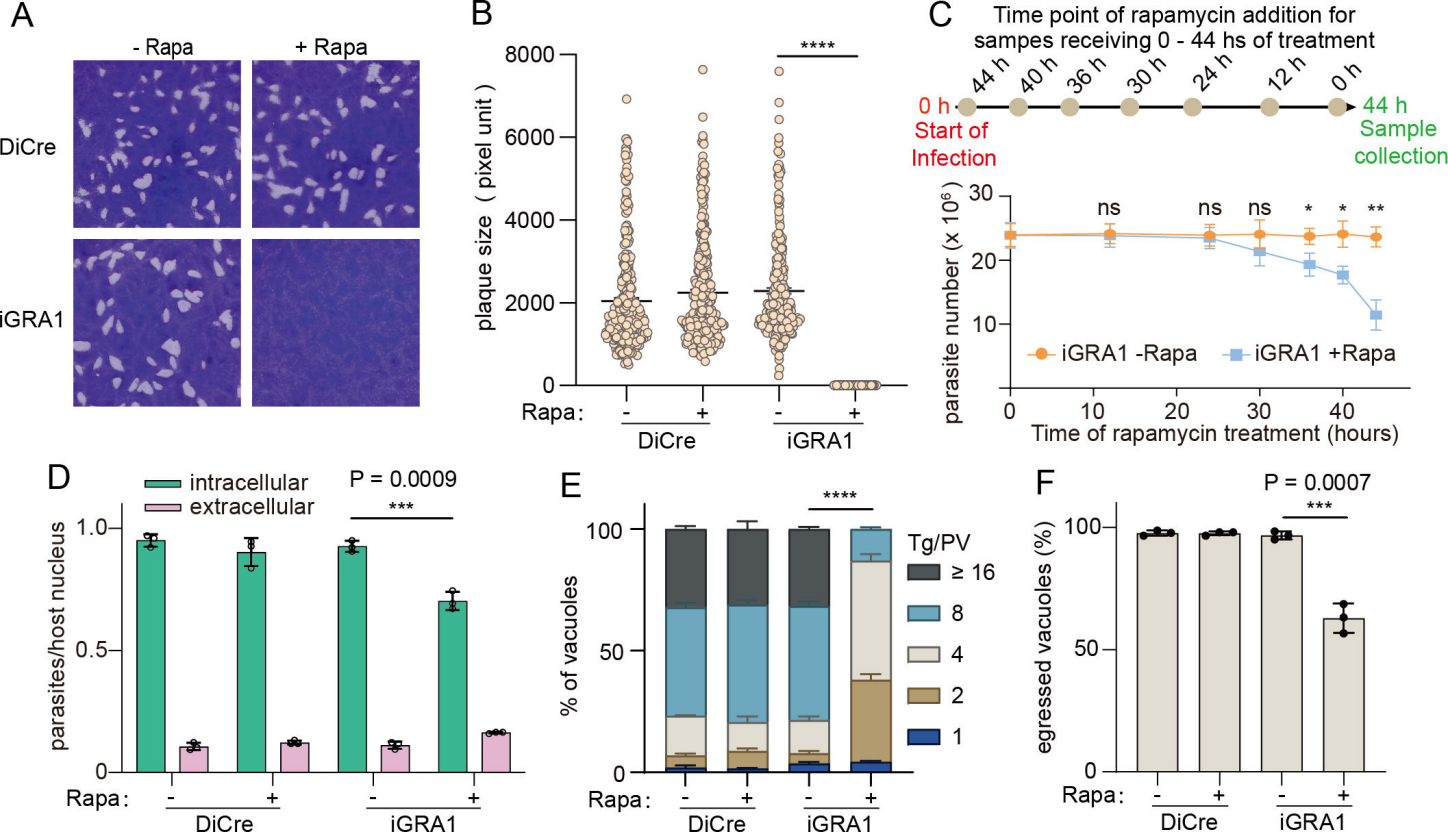
GRA1 is essential for the lytic cycle of *T. gondii* tachyzoites. (**A**) Plaque assay comparing parasite growth in the presence or absence of 50 nM rapamycin under normal growth conditions. (**B**) Quantification of the sizes of plaques from panel **A**. Mean ± SD of three independent experiments, ****P < 0.0001, unpaired two-tailed Student’s *t*-test. (**C**) Propagation of the iGRA1 strain treated with or without rapamycin. Purified tachyzoites of iGRA1 (2 × 10⁶) were used to infect HFF cells seeded in T25 flasks and cultured for 44 hours. During parasite growth, rapamycin was added to the cultures at different time points to a final concentration of 50 nM. Then, the samples were collected, and the number of parasites was counted. Mean ± SD of three independent experiments. *P < 0.05, **P < 0.01, unpaired two-tailed Student’s *t*-test. (**D**) Invasion efficiency of parasites treated with or without rapamycin (Rapa) for 36 hours. Mean ± SD of three independent experiments. ***P = 0.0009, unpaired two-tailed Student’s *t*-tests. (**E**) Intracellular replication assay of parasites with or without GRA1. The DiCre and iGRA1 strains were first treated with or without 50 nM rapamycin for 36 hours. Then, the parasites were collected to infect fresh HFF cells and cultured for 24 hours under the same pretreatment conditions. Subsequently, the samples were fixed, and the number of parasites in each PV was determined by IFA. Means ± SEM of three independent experiments, each with three replicates. ****P < 0.0001, two-way ANOVA followed by Tukey’s multiple comparison tests. (**F**) Calcium ionophore A23187 induced egress of parasites. Similar to the replication assay, parasites were first treated with or without rapamycin for 36 hours and then used to infect fresh HFF cells. Then, the parasites were cultured for 32 hours with or without rapamycin and then treated with 2 µM A23187 for 2 min to induce egress, which was determined by IFA staining of GRA7 to assess the integrity of PVs. Means ± SD of three independent experiments were graphed. ***P = 0.0007, unpaired two-tailed Student’s *t*-test.

To test the role of GRA1 during tachyzoite growth, a plaque assay was used to estimate the overall fitness of the GRA1 deletion mutant. Rapamycin treatment completely blocked plaque formation of the iGRA1 strain. In contrast, it did not affect the plaque formation of the parental strain DiCre (Fig. 2A and B), suggesting that GRA1 is crucial for tachyzoite growth. We also performed a parasite proliferation assay in which the iGRA1 strain was allowed to proliferate for 44 hours and rapamycin was added to the cultures at different time points to achieve a total rapamycin treatment time of 0–44 hours. Then, the samples were collected, and the number of parasites was counted under a microscope. The results showed that, with 36 hours of rapamycin treatment, the parasites started to display reduced proliferation, compared to the cultures without rapamycin treatment (Fig. 2C). Based on the rapamycin treatment time required to deplete GRA1 to undetectable levels (Fig. 1D), as well as the minimal duration of treatment time to see a proliferation defect (Fig. 2C), treating the iGRA1 strain with 50 nM rapamycin for 36 hours was used for subsequent experiments when GRA1 deletion mutants were needed.

The lytic cycle that contributes to plaque formation consists of multiple steps. To see which steps were affected by GRA1 deletion, the efficiency of host cell invasion, intracellular replication, and egress was examined (Fig. 2D through F). First, a two-color assay that distinguishes intracellular and extracellular parasites was used to assess the invasion efficiency of parasites. Treating the iGRA1 parasites with 50 nM rapamycin for 36 hours to deplete GRA1 reduced the invasion efficiency of parasites by more than 30% (Fig. 2D). To test the replication efficiency, iGRA1 or parental parasites were first treated with or without rapamycin for 36 hours. Then, the parasites were used to infect fresh host cells (HFF) and cultured for 24 hours (in the absence of rapamycin). Subsequently, the number of parasites in each PV was determined by IFA to estimate the parasites’ replication efficiency. While rapamycin treatment did not affect the replication of the parental strain DiCre, it significantly decreased the replication of iGRA1 parasites (Fig. 2E). In the calcium ionophore A23187-induced egress assays, treating the iGRA1 strain with rapamycin for 36 hours reduced the egress of parasites by 40% (Fig. 2F). Together, these results showed that GRA1 depletion reduced the efficiencies of all three major events (invasion, replication, and egress) during the lytic cycle of tachyzoites. It should be mentioned that the reduced invasion, replication, and egress of the GRA1 deletion mutant at this time point (36 hours after rapamycin treatment) were not due to the death of the parasites since these parasites were still able to replicate even after longer periods of growth (36 hours of rapamycin treatment followed by 24–48 h of growth), although the replication rates were significantly reduced compared to that without rapamycin treatment (Fig. S1). These results suggest critical roles of GRA1 during the lytic cycle of *Toxoplasma* in the type I strain RH.

### Deletion of GRA1 disrupted IVN integrity and altered parasite metabolism

**Fig 3:**
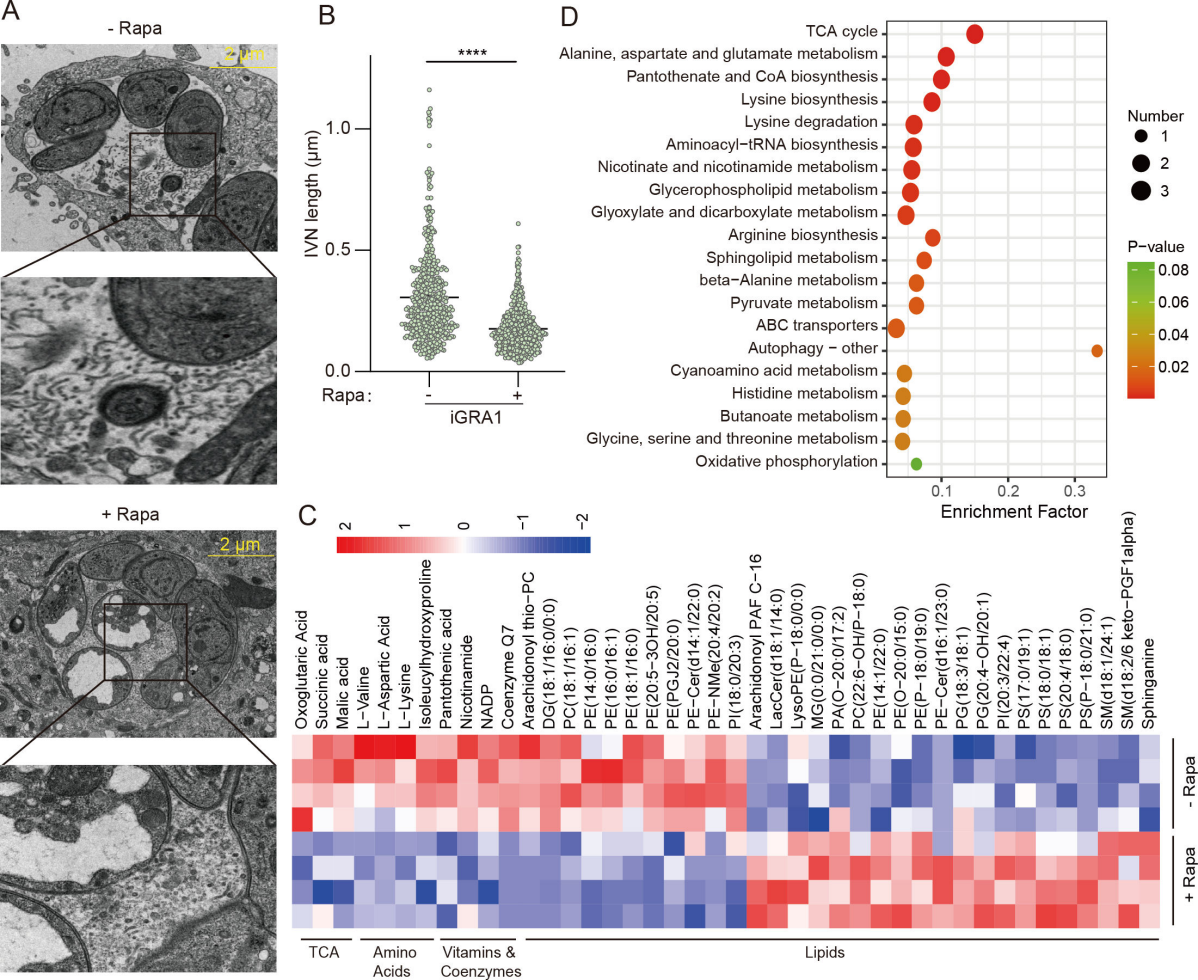
GRA1 is important for the structural integrity of the intravacuolar network (IVN) in PVs and metabolic homeostasis of the parasites. (**A**) Transmission electron microscopy analysis of IVN structures in iGRA1 parasites. Parasites were treated with (+ Rapa) or without (– Rapa) 50  nM rapamycin for 36 hours and then used to infect fresh HFF monolayers. After that, they were cultured for an additional 24 hours in normal medium (without rapamycin) before being processed for transmission electron microscopy to examine the IVN morphology. (**B**) Quantification of the lengths of IVNs observed by transmission electron microscopy. A total of >400 IVNs for each condition were measured. ****P < 0.001, Student’s *t*-test. (**C**) Heat map showing the abundance changes of selected metabolites in the iGRA1 strain after 44 hours of rapamycin treatment to deplete GRA1. (**D**) Metabolic pathway enrichment analysis of metabolites with significantly altered abundance after 44 hours of rapamycin treatment in the iGRA1 strain.

By transmission electron microscopy (TEM), previous work has shown that GRA1 is localized to both the PV space and the IVN (44). To see whether GRA1 has a role in the structure or function of IVN in the PV, we used TEM to compare the tubular structures in the PV before and after GRA1 deletion. The results indicated that rapamycin treatment of the iGRA1 strain indeed resulted in significant ultrastructural changes in the parasites. Notably, the tubular structures corresponding to IVN become much shorter after GRA1 deletion (Fig. 3A and B). Concomitantly, there was substantial accumulation of electron-dense material within the PV of the Δ*gra1* mutant. These ultrastructural alterations suggest that GRA1 has an important role in maintaining the morphology of the IVN. Previous studies have indicated that the IVN may play crucial roles in nutrient salvage from the host cells (34, 41, 46–48), although the detailed mechanisms are not yet known. The structural disruption of IVN by GRA1 deletion implies that GRA1 may affect the import of nutrients from host cells. To check this possibility, fresh tachyzoites of the iGRA1 strain treated with or without rapamycin for 44 hours were collected, and the metabolite levels were determined by LC/GC-MS. The results showed that GRA1 deletion indeed caused a major metabolic alteration in the parasites. The abundance of many metabolites, including amino acids, vitamins, and lipids, was significantly changed (Fig. 3C). KEGG pathway analysis of the metabolites with altered abundance showed that they were primarily enriched in amino acid metabolism, oxidative phosphorylation, nicotinate and nicotinamide metabolism, and pantothenate and CoA biosynthesis (Fig. 3D). We also performed similar metabolic analyses using the iGRA1 or the parental strain that were treated with or without rapamycin for 36 hours. The metabolic changes in the iGRA1 strain following 36 hours of rapamycin treatment were similar to those receiving 44 hours of treatment (Fig. S2). On the other hand, rapamycin treatment did not affect the metabolite levels in the parental strain DiCre (Fig. S2), suggesting that GRA1 was responsible for the metabolic changes in the GRA1 depletion mutant.

### Depletion of GRA1 impairs nutrient salvage and the integrity of the mitochondria and apicoplasts

**Fig 4:**
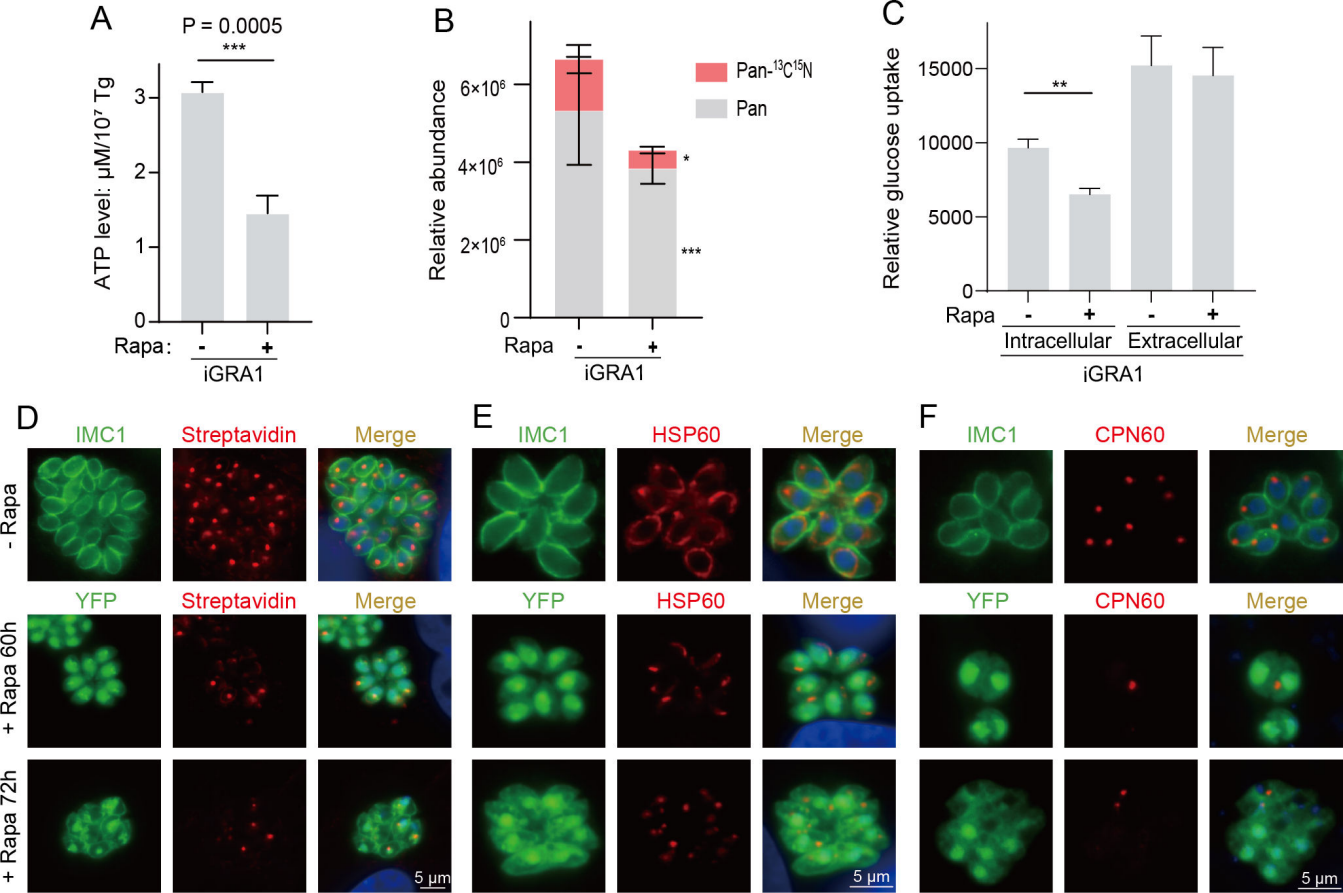
GRA1 is critical for the salvage of exogenous nutrients and to maintain the integrity of the mitochondrion and apicoplast organelles. (**A**) ATP levels in iGRA1 parasites treated with or without 50 nM rapamycin for 36 hours. Mean ± SD from three independent experiments. ***P = 0.0005, Student’s *t*-test. (**B**) Uptake of 13C15N-labeled pantothenate into intracellular iGRA1 parasites that were treated with or without 50 nM rapamycin. Means ± SEM of four independent experiments, *P < 0.05, ***P < 0.001, Student’s *t*-test. (**C**) Import of fluorescent glucose analog 2-NBDG into intracellular or extracellular iGRA1 parasites that were treated with or without 50 nM rapamycin. Means ± SEM of three independent experiments, **P < 0.01, Student’s *t*-test. (**D**) Protein biotinylation in iGRA1 parasites treated with or without 50 nM rapamycin, as detected by IFA using Alexa 594-conjugated streptavidin. (**E, F**) structural integrity of the mitochondrion (**E**) and apicoplasts (**F**) in iGRA1 parasites treated with or without rapamycin, as determined by IFA probing the organelle specific markers HSP60 and CPN60, respectively.

To further investigate the impact of GRA1 deletion on parasite metabolism, we first measured the total ATP levels in the parasites. The results showed that treating the iGRA1 strain with rapamycin for 36 hours reduced the cellular ATP levels by about 60% (Fig. 4A). The above metabolic analysis results revealed that GRA1 deletion led to reduced levels of various metabolites, including amino acids, pantothenate, and numerous metabolic intermediates. Furthermore, transmission electron microscopy observations indicated that GRA1 deletion disrupted the structure of the IVN, suggesting that GRA1 may play a role in maintaining IVN integrity, thereby facilitating nutrient uptake. Therefore, these findings prompted us to investigate whether GRA1 is involved in the uptake of nutrients, such as pantothenate, from host cells or the surrounding environment. To this end, the iGRA1 strain was first cultured in pantothenate-free medium (with or without 50 nM rapamycin) for 4 hours. Subsequently, stable isotope-labeled pantothenate (^13^C^15^N-Pan) was added to the cultures to a final concentration of 100 µM, and the parasites were cultured for 40 hours with or without rapamycin, as described before (51). Metabolites were then extracted, and the abundance of ^13^C^15^N-labeled and unlabeled pantothenate was quantified by liquid chromatography-mass spectrometry. The results indicated that the uptake of ^13^C^15^N-Pan reduced by roughly 30% after GRA1 deletion (Fig. 4B). The total concentration of pantothenate in the parasites was also reduced by 35% (Fig. 4B). These findings suggest that GRA1 might contribute to exogenous nutrient acquisition. To further check the nutrient scavenging role of GRA1, a fluorescent analog of glucose called 2-[N-(7-nitrobenz-2-oxa-1,3-diazol-4-yl) amino]−2-deoxy-D-glucose (2-NBDG) was used to test the ability of intracellular and extracellular parasites to absorb exogenous glucose. The iGRA1 parasites were first treated with or without rapamycin for 36 hours. Then, either a final concentration of 100 µM 2-NBDG was added to the intracellular cultures and incubated for 1 hour or the parasites were released from host cells and then incubated with 2-NBDG in extracellular medium for 30 min. Subsequently, the amount of 2-NBDG absorbed in each parasite was determined by flow cytometry. Similar to the ^13^C^15^N-Pan uptake assay, GRA1 deletion reduced overall intracellular 2-NBDG uptake by approximately 35% (Fig. 4C). However, the ability of extracellular parasites to take up glucose remained unaffected in the absence of GRA1, which is consistent with the role of GRA1 in the PV for nutrient salvage. To test the impact of GRA1 depletion on the uptake of additional nutrients, we indirectly tested the uptake of biotin by assessing the protein biotinylation levels in the parasites. The results indicated that depletion of GRA1 dramatically reduced protein biotinylation in the mitochondrion and apicoplasts (Fig. 4D), where most of the naturally biotinylated proteins are located (Fig. S3). Together, these results support the role of GRA1 in mediating nutrient uptake into parasites during intracellular growth. Some of these nutrients, like pantothenate, biotin, and nicotinamide, cannot be synthesized by the parasites and must be salvaged from the hosts. Given the key roles of these metabolites in generating cofactors like CoA that are essential for many metabolic reactions, their decreased supply upon GRA1 deletion caused systemic metabolic alterations, which could have additional consequences beyond metabolic failure. For example, when the structural integrity of the mitochondrion and apicoplasts was assessed by IFA, it was found that prolonged GRA1 depletion significantly impaired the structures of these organelles. By 60 hours post-rapamycin treatment, the intact staining pattern of the mitochondrial marker HSP60 became fragmented and spotty, which was further exacerbated in parasites treated with rapamycin for 72 hours (Fig. 4E). Similarly, without rapamycin treatment, each parasite of the iGRA1 strain had an apicoplast, as indicated by clear staining of the apicoplast-specific marker CPN60. Rapamycin treatment abolished CPN60 staining in many parasites (Fig. 4F), suggesting that the structural integrity of the apicoplast was compromised in the absence of GRA1.

### Depletion of GRA1 impairs carbon metabolism homeostasis

**Fig 5:**
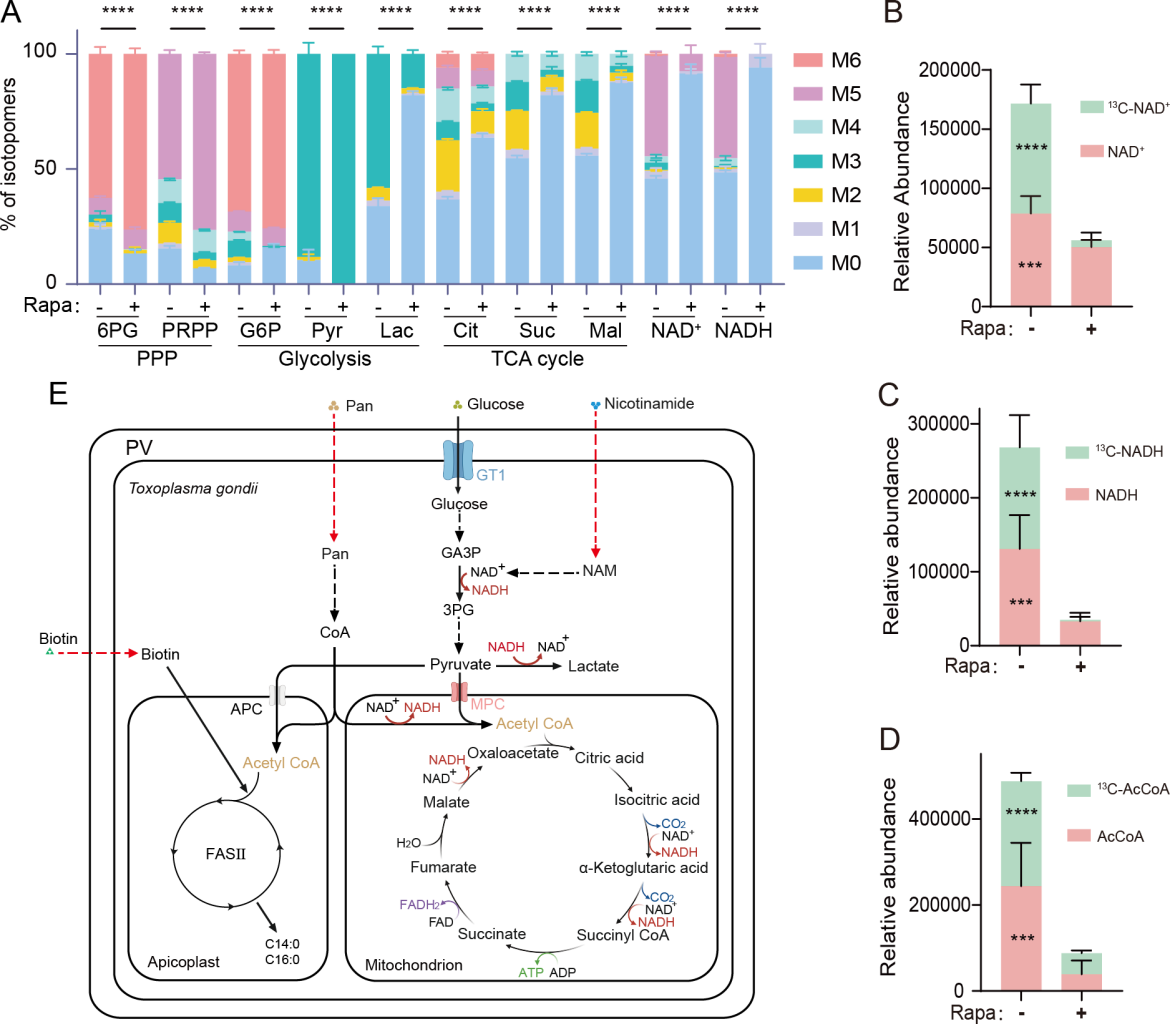
GRA1 depletion leads to impaired glucose catabolism in the parasites. (A) Metabolic labeling of iGRA1 parasites treated with or without 50 nM rapamycin for 36 hours. After rapamycin treatment, parasites were released from host cells and incubated in DMEM containing 8 mM ^13^C-glucose for 4 hours. Then, the metabolites were extracted, and the incorporation of 13C into different metabolites was analyzed by LC-MS. M0–M6 indicate the number of ^13^C incorporated into each metabolite. Mean ± SD of six independent experiments. ****P < 0.0001, two-way ANOVA. (B–D) Relative abundance of 13C-labeled and unlabeled NAD^+^ (B), NADH (C), and acetyl-CoA (D) in iGRA1 parasites treated with or without rapamycin. Data were derived from the same experiments described in (A). Mean ± SD from six independent experiments. Statistical comparisons between rapamycin-treated and untreated conditions were performed using Student’s *t*-test (****P  <  0.0001; ***P  <  0.005). (E) Metabolic pathways in *Toxoplasma*, highlighting the salvage of key exogenous nutrients (glucose, pantothenate (Pan), nicotinamide, and biotin) and glucose catabolism.

As mentioned above, depletion of GRA1 reduced the uptake of nutrients like pantothenate and biotin that are key for the biosynthesis of essential metabolic cofactors like CoA. As a consequence, GRA1 deletion might cause systemic metabolic alterations. To further estimate the metabolic disorders caused by GRA1 deficiency, we performed metabolic flux analyses by labeling the purified extracellular parasites with ^13^C-glucose and then monitoring the incorporation of ^13^C into different metabolites. The 2-NBDG uptake assay described above demonstrated that GRA1 deletion impaired glucose uptake in intracellular parasites but not in extracellular parasites. Therefore, we first treated the iGRA1 parasites with or without rapamycin for 36 hours. Then, the parasites were released from host cells by syringe passage and collected for ^13^C-glucose labeling. After incubating the parasites with 8 mM ^13^C-glucose for 4 hours, metabolites were extracted from the parasites, and ^13^C incorporation was quantified by LC-MS. The results found that GRA1 deletion dramatically reduced the flux of glucose to the TCA cycle (Fig. 5A). Interestingly, the labeling of lactate by ^13^C was also drastically reduced (Fig. 5A), suggesting the conversion of pyruvate to lactate was suppressed in the absence of GRA1. This is likely due to the reduced levels of NADH in the parasites due to decreased synthesis of NAD^+^ and NADH (Fig. 5A through C). In contrast to the TCA cycle, the flux of glucose to the pentose phosphate pathway seemed to be increased after GRA1 deletion (Fig. 5A). On the other hand, the level of acetyl CoA and the labeling of acetyl CoA with ^13^C were greatly decreased in the absence of GRA1 (Fig. 5D). Decreased levels and synthesis of acetyl CoA and NAD^+^/NADH may partially explain the reduced activities of the TCA cycle (Fig. 5E).

### GRA1 is required for the proper localization of dense granule proteins

**Fig 6:**
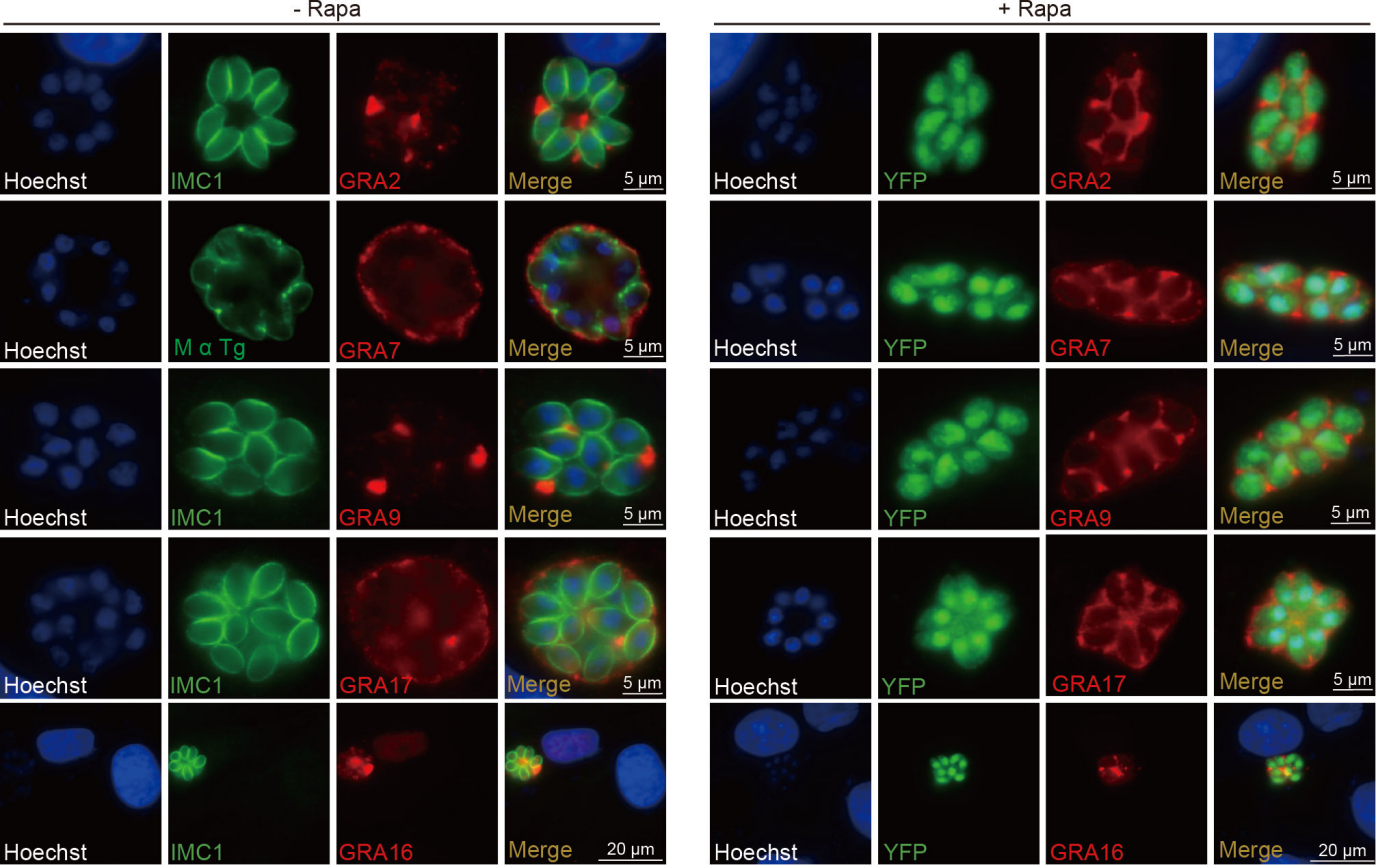
GRA1 is critical for the localization of dense granule proteins to the desired destinations. The iGRA1 strain was first treated with or without 50 nM rapamycin (Rapa) for 36 hours and then used to infect fresh HFF cells. The parasites were cultured for another 24 hours (without rapamycin) before immunofluorescence analyses to examine the localization of various dense granule proteins. GRA7 was detected using a rabbit polyclonal antibody, while all other dense granule proteins were probed by an HA antibody using iGRA1 lines with the corresponding GRA genes tagged with a 3*HA epitope. M α Tg: mouse anti-*Toxoplasma* IgG.

To investigate the underlying mechanisms associated with the metabolic and nutrient uptake defects of the GRA1 deletion mutant, we examined whether the loss of GRA1 affected the localization of other dense granule proteins that were targeted to different cellular compartments after release from the dense granules. Immunofluorescence assays (IFAs) revealed that the subcellular localization of many GRA proteins was altered in the absence of GRA1. They failed to localize to the final destinations; instead, they aggregated in the PV around the parasite surface. GRA7 and GRA17 were localized to the PVM of parental parasites, but were in the PV close to each parasite after GRA1 deletion (Fig. 6). Similarly, GRA2 and GRA9 were in the PV space with a distance to the parasite surface, yet they became very close to the parasite surface in the absence of GRA1 (Fig. 6). GRA16 was secreted to the host nucleus from the parasite in the presence of GRA1 but failed to do so and was retained in the PV after GRA1 deletion (Fig. 6). These results seem to suggest that GRA1 is needed for secretory GRA proteins to traffic beyond the PV after being discharged from parasite dense granules. Interestingly, GRA17 is critical for nutrient uptake by forming a pore on the PVM to allow the entry of nutrient molecules. Mislocalization of proteins like GRA17 may explain the nutrient salvage defects of the Δ*gra1* mutant. To assess whether the mislocalization of dense granule proteins was due to reduced expression or secretion levels in the Δ*gra1* mutant, we detected the protein levels of GRA2 and the micronemal protein MIC2 in both the pellet (in parasites) and supernatant (secreted) fractions after ethanol-induced secretion. The results demonstrated that GRA1 deletion did not affect the secretion or expression levels of GRA2 or MIC2 (Fig. S4). These findings suggest that GRA1 associated with the IVN plays a pivotal role in the trafficking of secretory GRA proteins from PV to their final destinations, without affecting their expression or secretion into PV from the dense granules.

### Depletion of GRA1 promotes bradyzoite differentiation under stress conditions

**Fig 7:**
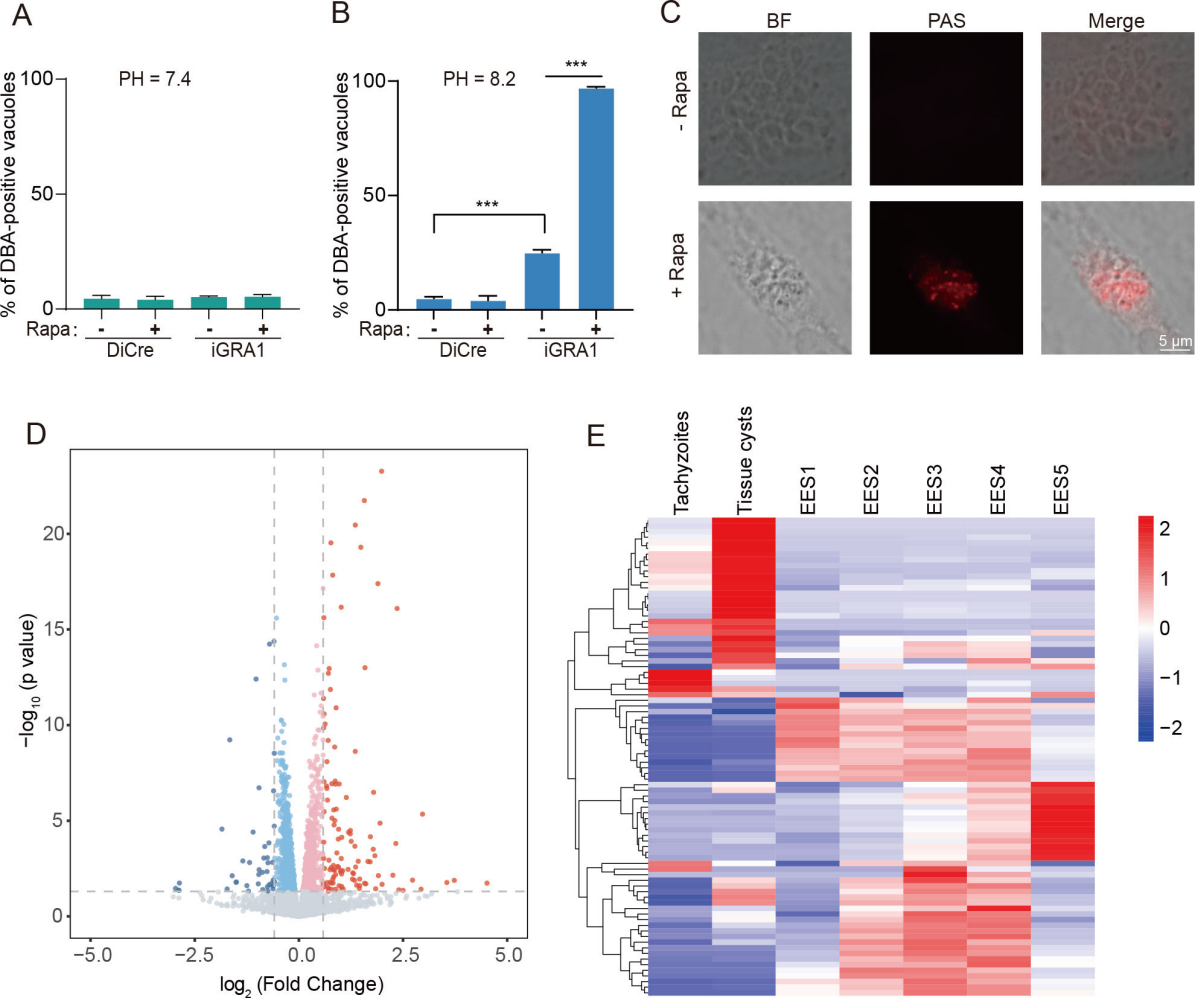
Depletion of GRA1 promotes bradyzoite transition under stress conditions. (**A**) Percentage of DBA-positive vacuoles in parasites under normal growth conditions (pH = 7.4, 5% CO_2_). Parasites were first treated with or without 50 nM rapamycin for 36 hours and then used to infect fresh HFF cells. Parasites were cultured for another 48 hours (without rapamycin) before DBA staining. (**B**) Percentage of DBA-positive vacuoles in parasites cultured under alkaline (pH = 8.2, ambient CO_2_) growth conditions. Parasites were first treated with or without rapamycin for 36 hours under normal growth conditions. Then, the parasites were used to infect fresh HFF cells and cultured in alkaline medium for 72 hours (without rapamycin) before DBA staining. Means ± SD of three independent experiments, ***P < 0.001, unpaired two-tailed Student’s *t*-test. (**C**) Amylopectin accumulation in the GRA1 depletion mutants under alkaline growth conditions, as shown by PAS staining. Samples were processed as in (**B**) before PAS staining. (**D**) Volcano plot illustrating the gene expression changes in the iGRA1 strain after 44 hours of rapamycin treatment in normal growth medium, as determined by RNA-Seq. Fold change (+rapamycin/–rapamycin) of the mRNA level of each gene was used to generate the plot. Data were from *n* = 4 independent experiments. (**E**) Heatmap showing the expression patterns of genes upregulated in the GRA1 depletion mutants during the life cycle of wild-type parasites. Data were obtained from ToxoDB.

In many cases, slowing down parasite growth often promotes the tachyzoite-to-bradyzoite transition. To check whether this is the case for the Δ*gra1* mutant, we first examined the differentiation of the parasites under normal conditions (pH 7.4). The results showed that treating the iGRA1 strain with rapamycin to deplete GRA1 expression had no impact on parasite differentiation (Fig. 7A), with a very low percentage of the PVs being DBA-positive. Next, we cultured the parasites under alkaline conditions (pH 8.2, ambient CO_2_) to induce bradyzoite conversion and determined the bradyzoite transition rate by DBA staining. The DiCre strain was constructed from the type I strain RH, which lost the capacity to complete the life cycle and rarely differentiate into bradyzoites even under stress conditions. Consistently, the DiCre strain barely formed bradyzoites under alkaline conditions (Fig. 7B). In contrast, the iGRA1 strain readily formed bradyzoites under these conditions, and about 25% of the PVs were DBA^+^ even without rapamycin treatment (Fig. 7B), significantly higher than that in the parental strain DiCre. This is likely due to the fact that the expression of GRA1 in the iGRA1 strain was driven by the tubulin promoter, as compared to the endogenous GRA1 promoter in the DiCre strain. With rapamycin to delete GRA1, nearly 100% of the iGRA1 parasites switched to bradyzoites under alkaline conditions (Fig. 7B). One hallmark of the mature bradyzoites encysted in cysts is that they accumulate large amounts of amylopectin. PAS staining was used to estimate the abundance of amylopectin in iGRA1 parasites cultured under alkaline conditions. The results showed that rapamycin-treated parasites displayed strong PAS staining, which was hardly detected in the absence of rapamycin treatment (Fig. 7C). These results further support that GRA1 deletion increased bradyzoite differentiation under stress conditions. The increased rates of bradyzoite conversion in GRA1-deficient mutants in the alkaline medium allowed us to check the trafficking of GRA proteins in bradyzoites. For this purpose, we tagged the endogenous *MAG1* gene (which has higher expression in bradyzoites than in tachyzoites) in the iGRA1 strain with a triple HA tag. Under alkaline conditions, MAG1-3*HA exhibited two types of localization in the iGRA1 strain without rapamycin treatment: within the PV when the expression was low (indicative of tachyzoites) and in the cyst wall when the expression was high (indicative of bradyzoites), consistent with the observation that about 25% of the iGRA1 parasites converted to bradyzoites under alkaline conditions (Fig. 7B). Interestingly, with rapamycin to deplete GRA1, the iGRA1/MAG1-3*HA strain cultured in the alkaline medium exhibited high expression levels of MAG1-3*HA, but it did not localize to the cyst wall and stayed in PV close to the parasites instead (Fig. S5). Again, these results further support that GRA1 is needed for the trafficking of GRA proteins beyond the PV to final destinations in both tachyzoites and bradyzoites.

Increased rates of bradyzoite transition of the GRA1 deletion mutant prompted us to check the gene expression changes upon GRA1 deletion. RNA-Seq analyses on the iGRA1 strain treated with or without rapamycin in normal medium revealed that GRA1 depletion resulted in altered transcription (fold change ≥ 2, *P* value < 0.05) of 158 genes (Fig. 7D), 115 of which were upregulated and 43 were downregulated after GRA1 deletion. For a mutant with such a strong growth defect, this gene expression change is not considered dramatic, suggesting that GRA1 probably does not work by manipulating gene expression. Interestingly, among the limited number of differentially expressed genes, 24% of the upregulated genes were specifically expressed during the bradyzoite stage and 50% were specific to the merozoite stage (Fig. 7E). Given the localization of GRA1 in the PV, a direct role in gene expression regulation is not expected. Instead, the gene transcription changes in the GRA1 depletion mutant were likely caused by the reduced nutrient uptake from the host cells, which might act like a stress factor to initiate parasite development.

### GRA1 is critical for the growth of the type II strain ME49 both *in vivo* and *in vitro*

**Fig 8:**
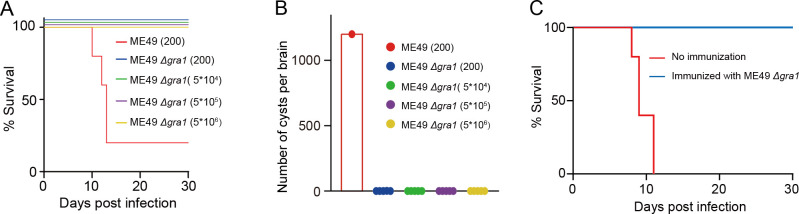
GRA1 is important for the growth and virulence of the type II strain ME49. (**A**) Survival curves of KM mice infected with different doses of *Toxoplasma* tachyzoites (200, 5*10^4^, 5*10^5,^ or 5*10⁶ tachyzoites/mouse, *n*  =  5 mice for each strain or dose). (**B**) The number of *Toxoplasma* cysts in the brains of mice survived in the virulence tests in panel **A**. (**C**) ME49 Δ*gra1* immunization protects mice from lethal *Toxoplasma* infection. KM mice were first immunized with ME49 Δ*gra1* (10^4^ per mouse) (or DMEM as a no immunization control). Thirty days post-vaccination, all mice were challenged with 10⁴ tachyzoites of the ME49 strain by intraperitoneal injection. Survival of mice was then monitored daily for 30 days.

The results described above clearly show that GRA1 has a crucial role for tachyzoite growth in the type I strain RH. Attempts from another group to knock out this gene in RH failed (52), which is consistent with its critical role for parasite growth. However, more recently, the same group successfully knocked out GRA1 in the type II strain PRU (53). Although the growth phenotypes of the PRU Δ*gra1* mutant were not fully analyzed, the construction of a clean knockout suggests that GRA1 is dispensable in PRU. To further test the role of GRA1 in type II strains, we tried to delete GRA1 in ME49. First, we generated an ME49/loxP-GRA1 strain to flox the endogenous GRA1 gene (Fig. S6A), just like what we did in the RH strain. Then, the floxed GRA1 was deleted through a plasmid expressing the Cre recombinase (Fig. S6A). The resulting ME49 Δ*gra1* strain was confirmed by diagnostic PCR and Western blotting, which verified the deletion of *GRA1* (Fig. S6B and C). Intracellular replication and plaque assays showed that GRA1 deletion significantly reduced the growth and replication of ME49 (Fig. S6D and E), suggesting critical but non-essential roles of GRA1 in the type II strain ME49, which is consistent with what was observed in PRU. To examine the impact of GRA1 deletion on bradyzoite differentiation in ME49, we induced bradyzoite formation using alkaline conditions, and the results indicated that, as observed in the RH strain, GRA1 deletion in ME49 also resulted in higher rate of bradyzoite conversion (Fig. S6F). The construction of a clean GRA1 knockout strain in ME49 also allowed us to test the role of GRA1 in parasite virulence and cyst development during *in vivo*. KM mice were infected intraperitoneally with various doses of ME49 or ME49 Δ*gra1* tachyzoites, and the symptoms of infected mice were monitored daily. Consistent with *in vitro* growth defects, ME49 Δ*gra1* displayed dramatically reduced virulence in mice (Fig. 8A). At the infection dose of 200 tachyzoites per mouse, the parental ME49 strain caused 80% mortality within 12 days. In contrast, even at doses as high as 5 × 10^6^ tachyzoites per mouse, ME49 Δ*gra1* did not cause mortality (the infected mice did undergo seroconversion, suggesting ME49 Δ*gra1* could establish infection in mice), indicating substantial attenuation of virulence. Thirty days after infection, the number of *Toxoplasma* cysts in mice that survived the infection was counted. The results showed that no cysts were found in mice infected with ME49 Δ*gra1* (Fig. 8B), likely due to the poor growth of these parasites. To evaluate the vaccine potential of ME49 Δ*gra1*, mice were immunized with 1  ×  10^4^ ME49 Δ*gra1* tachyzoites or left unvaccinated. Thirty days later, each mouse was challenged with 1  ×  10^4^ tachyzoites of the ME49 strain, and the survival of mice was monitored. All unvaccinated mice died 11 days after ME49 challenge, whereas 100% of the mice immunized with ME49 Δ*gra1* survived the challenge (Fig. 8C). These results demonstrate that ME49 Δ*gra1* could establish subclinical infections in mice and elicit protective immunity. Together, these data suggest that although GRA1 is not essential in the type II strain ME49, it is critical for parasite growth both *in vitro* and *in vivo*.

## DISCUSSION

When replicating in host cells, *Toxoplasma* parasites are enclosed in the PV that is non-fusogenic with the host lysosome. The PV represents a barrier for direct communications between the parasite and the host cell. Therefore, the parasites have evolved sophisticated mechanisms to bypass these limitations. They secrete diverse effector proteins to the PV, PVM, and host cells to manipulate host-pathogen interactions for the benefits of the parasites. The functions of the vast majority of these secretory proteins await full dissection. Notably, GRA1 was the first GRA protein reported 35 years ago, but its precise function has never been determined. In this study, we used genetic and cell biological approaches to investigate the role of GRA1, and the results show that GRA1 is critical for the parasite to scavenge nutrients from host cells. By localizing to the IVN and PV, GRA1 is needed for the structural integrity of the IVN. Conditional deletion of GRA1 resulted in a shortened and morphologically altered IVN, as well as mislocalization of other GRA proteins, including GRA17 that normally localizes to the PVM to form a channel to facilitate host nutrient uptake. Therefore, GRA1-deficient parasites suffered from reduced metabolic activities and impaired growth, concomitant with increased tendency of bradyzoite conversion.

As an obligate intracellular parasite, *Toxoplasma* strictly depends on host cells for nutrient supply. Although it has decent capacity to synthesize many metabolites and displays great metabolic flexibility, *Toxoplasma* needs to take up key nutrients from host cells, such as carbon sources, vitamins, and inorganic iron. For this purpose, the parasite has evolved sophisticated strategies, one of which is the extensive modification of the PV and PVM. The PVM contains both inward-extending and outward-extending membranous tubules, which are called extra-vacuolar vesicular network (EVN) and IVN, respectively. Both EVN and IVN in *Toxoplasma* contain GRA proteins, but their functions are not clear. IVN is clearly involved in the import of host nutrients into the PV, as demonstrated by mutants lacking GRA2 or GRA6, which are key to the nanotubular architecture of the IVN. GRA1 is also localized to the IVN, and it is key for the structural integrity of the IVN. Consistent with the role of IVN in nutrient salvage, Δ*gra1* mutants had reduced glucose and pantothenate salvage capacity, which led to decreased metabolic activities and slower growth. However, the phenotypes of the Δ*gra1* mutants are not the same as those lacking GRA2 or GRA6. Unlike Δ*gra1*, the GRA2 or GRA6 null mutants seem to have nearly normal growth and virulence, despite having strong defects in IVN formation. The underlying reasons for the phenotypic differences between Δ*gra1* and Δ*gra2* or Δ*gra6* mutants are currently unknown. These results suggest that the defects in IVN structure may not be the only reason for the poor growth of the Δ*gra1* mutant.

In addition to the IVN, GRA1 also localizes to PV space, whereas GRA2 and GRA6 strongly associate with the PVM. Moreover, the trafficking of many GRA proteins to their final destinations was interrupted in the Δ*gra1* mutant. These include GRA17 that forms the nutrient import pore on the PVM (23), as well as GRA7, which has a role in facilitating the internalization of host vesicles into the PV (41). These defects probably explain the nutrient salvage and metabolic defects of the Δ*gra1* mutant. It is not clear whether the Δ*gra2* or Δ*gra6* mutants have the same defects in GRA protein trafficking. Interestingly, the trafficking of GRA2 was also altered in the Δ*gra1* mutant as it failed to reach far from the parasite surface after discharge from the dense granules. Since GRA2 is required for the curved and elongated tubular structures of the IVN, mistargeting of GRA2 may explain the shorter IVN seen in the Δ*gra1* mutant. In addition to the GRA proteins in the INV or on the PVM, GRA1 is also required for the trafficking of GRA 16 to the nuclei of infected host cells. Together, these results suggest that GRA1 is needed to mobilize the GRA proteins from the site of secretion to their final destinations. But the exact mechanism is currently unknown and deserves further investigations.

## MATERIALS AND METHODS

### Parasite and host cell culture

All the genetically modified strains used in this study were derived from the parental strains RH Δ*ku80* Δ*hxgprt*-DiCre-T2A (DiCre) (50) or ME49, which were cultured in HFF (ATCC, VA, USA) monolayers in Dulbecco’s modified Eagle’s medium supplemented with 2% fetal bovine serum (Gibco USA), 100 unit/mL penicillin, 100 µg/ml streptomycin and 2 mM glutamine. Rapamycin (Aladdin, China) at a final concentration of 50 nM was used to induce GRA1 excision in the iGRA1 strain.

### Construction of plasmids and transgenic parasite strains

All plasmids used in this study and the methods for their construction are listed in Table S1. All primers used in this study are listed in Table S2 and were synthesized by Tsingke Biotechnology Limited (Beijing, China). Locus-specific CRISPR constructs were generated by replacing the UPRT-targeting guide RNA in the pSAG1-Cas9-U6-sgUPRT plasmid with gene-specific guide RNAs, following a previously described protocol (54). Other plasmids were constructed using the Clon-Express MultiS One Step Cloning Kit (Vazyme Biotech, China). The pUC19-5H-Tub-LoxP-GRA1-LoxP-YFP-HXGPRT was constructed by recombining the fragments of LoxP-YFP-HXGPRT, Tub-LoxP, 5′ and 3′ homologous arms, and CDS of GRA1 into the plasmid pUC19.

The iGRA1 strains were constructed by co-transfecting the 5H-Tub-LoxP-GRA1-LoxP-YFP-HXGPRT fragment and the GRA1 targeting CRISPR plasmid into the DiCre strain. Transfectants were selected with 25 µg/mL mycophenolic acid (MPA) and 50 µg/mL xanthine (Sigma-Aldrich, USA). Single clones were isolated by limiting dilution and subsequently screened by diagnostic PCRs (primers are listed in Table S2) and IFA.

### Immunofluorescent assays (IFAs) and Western blotting

IFA assays and Western blotting were performed according to previously described protocols (55). The primary antibodies used in this study include mouse anti-Ty monoclonal antibody (the BB2 clone of anti-Ty1, provided by Dr. David Sibley, Washington University in St. Louis, USA), rabbit anti-TgALD polyclonal antibody, rabbit anti-TgIMC1 polyclonal antibody (provided by Dr. Qun Liu from China Agricultural University, China), rabbit anti-HSP60, rabbit anti-CPN60 (provided by Dr. Honglin Jia at Harbin Veterinary Research Institute in China), and mouse anti-HA (the TANA2 clone of anti-HA, MBL International Corporation, Japan). Alexa Fluor 488-conjugated goat anti-mouse IgG, Alexa Fluor 594-conjugated goat anti-rabbit IgG, Alexa Fluor 488-conjugated goat anti-rabbit IgG, and Alexa Fluor 594-conjugated goat anti-mouse IgG (Fisher Scientific, USA) were used to detect primary antibodies. HRP-conjugated goat anti-rabbit and goat anti-mouse secondary antibodies (for WB), followed by detection with BeyoECL Moon Kit (Beyotime, China). IFA images were acquired from the Olympus FluoView FV1000 Confocal Microscope (Olympus Life Science, Japan) or Olympus BX53 Microscope (Olympus Life Science, Japan) equipped with an AxioCam 503 mono camera (Zeiss, Germany). The Western blots were scanned by an Amersham Typhoon 5 imager (GE Healthcare, UK).

### Phenotypic analyses

The plaque, invasion, egress, and replication assays were performed as previously described (56, 57), with a slight modification. The DiCre or iGRA1 strains were treated with or without 50 nM rapamycin for 36 hours before harvesting parasites for those assays. For the plaque assay, 200 tachyzoites per well were used to infect HFF monolayers seeded in six-well plates and cultured at 37°C with 5% CO_2_ for 7 days. The plaques were then visualized by crystal violet staining. For the invasion assay, pretreated parasites (5 × 10⁶) were inoculated onto confluent HFF cells seeded on coverslips and allowed to invade for 30 minutes at 37°C. Subsequently, the noninvaded parasites were washed away, and the rest were subjected to a two-color staining assay to distinguish extracellular (stained by pig anti-*Toxoplasma* antibody before permeabilization) from total parasites (marked by anti-ALD or YFP autofluorescence). Invasion efficiency was calculated as the ratio of intracellular parasites to host nuclei across 30 fields. For the parasite replication assay, pretreated parasites were used to infect fresh HFF cells seeded on coverslips and cultured for 24 hours (without rapamycin). The samples were then fixed and stained with anti-ALD or YFP. The number of parasites in each PV was counted in at least 150 vacuoles for each condition. For the egress assay, pretreated parasites (1 × 10⁶) were purified to infect HFF cells and cultured for 32 hours (without rapamycin). Then, A23187 at a final concentration of 2 µM was added to the cultures and incubated for 2 minutes to induce egress. Subsequently, the samples were fixed and stained with anti-GRA7 to check the integrity of PVs. Egress efficiency was determined by the percentage of egressed PVs.

### Transmission electron microscopy

HFF monolayers were cultured in T75 flasks to confluence and then infected with iGRA1 treated with or without rapamycin for 36 hours. Infected cells were incubated for 24 hours at 37°C. After incubation, cells were scraped, collected by centrifugation at 2,000 rpm for 10 minutes, and fixed in 0.25% (wt/vol) glutaraldehyde in 0.1 mol/L PBS at 25°C for 4 hours. Following fixation, cells were centrifuged at 2,000 rpm for 10 minutes, washed with PBS, and post-fixed in 1% (wt/vol) osmium tetroxide at room temperature (57, 58). The samples were then dehydrated through a graded ethanol series, embedded in resin, and polymerized. Ultrathin sections (60–70 nm) were cut using a UC6 Ultramicrotome (Leica, Germany), stained with 2% (vol/vol) uranyl acetate, and examined under a Hitachi H-7650 TEM at 80 kV to assess the morphological changes.

### Bradyzoite differentiation assay

Bradyzoite differentiation was induced *in vitro* using an established protocol (59, 60). Briefly, HFF cells grown on coverslips were infected with freshly purified tachyzoites for 1 hour under standard culture conditions. After removing uninvaded parasites by PBS washes, infected cells were cultured for 72 hours in alkaline medium (RPMI 1640 supplemented with 50 mM HEPES, 1% fetal bovine serum, pH 8.2, and ambient CO_2_) (59, 60). To assess the differentiation rate, samples were fixed with 4% paraformaldehyde, permeabilized with 0.1% Triton X-100, and analyzed by immuno-staining. Bradyzoite conversion was detected by labeling with rabbit anti-TgALD antibody (secondary antibody: Alexa-488 conjugated goat anti-rabbit IgG) and rhodamine-labeled Dolichos biflorus agglutinin (DBA) (Vector Laboratories, USA). Samples were visualized using an FV1000 LSCM confocal laser scanning microscope (Olympus, Japan). Bradyzoite differentiation efficiency was quantified by calculating the ratio of DBA-positive vacuoles to TgALD-positive vacuoles. All experiments were performed in triplicate.

### Untargeted metabolomics by LC-MS

*Toxoplasma* tachyzoites were cultured for 44 hours with or without rapamycin. After washing with pre-cooled PBS, parasites were harvested by cell scraping and syringe passage, followed by filtration through a 3 µm polycarbonate membrane. Parasites were pelleted by centrifugation (500 × *g*, 10 min). Metabolites were extracted with methanol: water (4:1, vol/vol) and chloroform, using L-2-chlorophenylalanine as an internal standard. Samples were subjected to ultrasonication, centrifugation (13,000 rpm, 4°C), and freeze-drying. Reconstitution was performed with methanol: water (1:4, vol/vol), followed by ultrasonication and another centrifugation. Supernatants were filtered (0.22 µm) and transferred to LC vials for analysis using a Dionex Ultimate 3000 UPLC system coupled with a TSQ Quantiva Ultra triple-quadrupole mass spectrometer (Thermo Fisher). Metabolites were separated on a Synergi Hydro-RP column with a binary mobile phase system (water + 10 mM tributylamine, methanol) under a 25 minute gradient. Data were acquired in the SRM mode with positive-negative ion switching. Source parameters included a capillary temperature of 350°C, heater temperature of 300°C, and source voltages of 3,500 V (positive) and 2,500 V (negative). Quality control samples ensured analytical reliability.

### Untargeted metabolomics by GC-MS

*Toxoplasma gondii* tachyzoites were collected as described for LC-MS. Metabolites were extracted using a methanol: chloroform mixture (4:1, vol/vol) with L-2-chlorophenylalanine as an internal standard. Samples were subjected to ultrasonication, centrifugation (13,000 rpm, 4°C), and freeze-drying. A pooled sample from all extracts served as a quality control (QC) reference. Dried samples were derivatized by adding 80 µL of 15 mg/mL methoxylamine hydrochloride in pyridine, vortexing, and incubating at 37°C for 90 minutes. Next, 80 µL of BSTFA (with 1% TMCS) and 20 µL of n-hexane were added, followed by vortexing and incubation at 70°C for 60 minutes.

Derivatized samples were analyzed on an Agilent 7890B GC system coupled to a 5977A MSD using a DB-5MS capillary column. Helium was used as the carrier gas at 1 mL/min. The oven temperature was programmed from 60°C to 305°C in multiple steps. The MS system operated in the full-scan mode (*m*/*z* 50–500), with a solvent delay of 5 minutes. Injector, ion source, and quadrupole temperatures were set to 260°C, 230°C, and 150°C, respectively. QC samples were injected regularly to assess data reliability.

### Pantothenate uptake assay

Pantothenate-free medium was custom-prepared based on the PM150210 formulation, excluding calcium pantothenate. ^13^C^15^N-pantothenate was purchased from Anplel (Cat# 356786-94-2). The iGRA1 tachyzoites were inoculated into confluent HFF monolayers, cultured in pantothenate-free DMEM (Pricella, China) with or without 50 nM rapamycin for 4 hours. Subsequently, ^13^C^15^N-pantothenate (ANPEL Laboratory Technologies, China) at the final concentration of 100 µM was added, and the parasites were cultured for an additional 40 hours. Prior to sample collection, cells were washed five times with PBS to remove residual pantothenate. Parasites were then released from host cells by passing through 27G needles a couple of times, purified by filtration through 3 µm membranes, and collected by centrifugation at 500 × *g* for 10 minutes. Subsequently, pantothenate was extracted from the parasites and subjected to LC-MS analysis.

### Glucose uptake assay

For the glucose uptake of intracellular parasites, freshly egressed iGRA1 tachyzoites were inoculated into confluent HFF monolayers and treated with or without 50 nM rapamycin for 32 hours in normal DMEM. Then, the growth medium was replaced with glucose-free DMEM (Pricella, China) with or without 50 nM rapamycin to starve the cells for 4 hours. Subsequently, 2-NBDG (Beyotime Biotechnology, China) at the final concentration of 100 µM was added and incubated for 1 hour. The samples were then washed five times with PBS to remove residual 2-NBDG, and the parasites were released from host cells by needle passage. The parasites were purified by filtration through 3 µm membranes, resuspended in PBS, and analyzed by a CytoFLEX LX flow cytometry (Beckman Coulter Life Sciences, USA). For the glucose uptake of extracellular parasites, the iGRA1 parasites were first treated with or without 50 nM rapamycin for 36 hours of incubation. Then, the parasites were collected, purified, counted, resuspended in 1 mL of glucose-free DMEM containing 100 µM 2-NBDG, and incubated at 37°C for 30 minutes. Subsequently, the parasites were collected by centrifugation at 500 × *g* for 10 minutes and washed twice with PBS. Finally, the parasites were resuspended in 500 µL PBS and analyzed by flow cytometry.

### RNA-seq

Total RNA was extracted using the TRIzol reagent (Invitrogen, USA) following the manufacturer’s protocol. RNA purity and concentration were measured with a NanoDrop 2000 spectrophotometer (Thermo Scientific, USA), and RNA integrity was assessed using the Agilent 2100 Bioanalyzer (Agilent Technologies, USA). Libraries were prepared using the VAHTS Universal V6 RNA-seq Library Prep Kit and sequenced on the Illumina NovaSeq 6000 platform, generating 150 bp paired-end reads. Sequencing and analysis were conducted by OE Biotech Co., Ltd. (Shanghai, China). Raw reads were processed with *fastp* to trim adapters and remove low-quality reads, resulting in clean reads, which were then aligned to the GT1 reference genome using *HISAT2* (61). Gene expression levels were quantified using HTSeq-count, and FPKM values were calculated. Principal component analysis (PCA) was performed using R (v 3.2.0) to assess sample clustering and variability. Differential expression analysis was conducted using DESeq2, with an adjusted *P*-value (Q-value) ≤0.05 and fold change ≥2 as thresholds for identifying significantly differentially expressed genes. Each condition was independently prepared and analyzed with three biological replicates to ensure data robustness.

### Virulence tests and cyst formation *in vivo*

Freshly egressed tachyzoites were collected, purified by filtration through membranes with a pore size of 3.0 µm, and resuspended in PBS as previously described (62). Subsequently, they were used to infect KM mice via intraperitoneal injection. The symptoms of infected mice were monitored daily for 30 days. Then, the surviving mice were euthanized; blood samples were collected for ELISA analysis (63), and brain tissues were homogenized for immunofluorescence assay (IFA) with FITC-DBA staining to enumerate brain cysts.

### Statistical analyses

Except for the RNA-Seq data, all other results were analyzed with the Prism 10 software (San Diego, CA, USA). Statistical analyses were performed by unpaired two-tailed Student’s *t* test or two-way ANOVA with Tukey’s multiple comparisons post-tests, as indicated in the figure legends. All experiments were performed at least three times.

## Data Availability

The RNA-Seq data sets generated in this study have been deposited to the GEO database with the accession number GSE280122.

## SUPPLEMENTAL MATERIAL

Figure S1 mbio.01242-25-s0001.pdf GRA1 is critical for rapid replication of *Toxoplasma* tachyzoites.

Figure S2 mbio.01242-25-s0002.pdf Metabolic changes in the iGRA1 strain after 36 hours of rapamycin treatment.

Figure S3 mbio.01242-25-s0003.pdf Protein biotinylation in iGRA1 parasites determined by IFA.

Figure S4 mbio.01242-25-s0004.pdf Secretion of microneme proteins and dense granule proteins by extracellular parasites of the iGRA1 strain treated with or without rapamycin for 36 hours.

Figure S5 mbio.01242-25-s0005.pdf Alteration of MAG1 localization after GRA1 depletion under alkaline growth conditions.

Figure S6 mbio.01242-25-s0006.pdf GRA1 is important for the growth of the type II strain ME49.

Table S1 mbio.01242-25-s0007.docx Plasmids used in this study.

Table S2 mbio.01242-25-s0008.docx Primers used in this study.

ASM does not own the copyrights to Supplemental Material that may be linked to, or accessed through, an article. The authors have granted ASM a non-exclusive, world-wide license to publish the Supplemental Material files. Please contact the corresponding author directly for reuse.
