# Supplementary material for: The critical role of Toxoplasma gondii GRA1 in nutrient salvage
Source: mBio. 2025 Jun 27;16(8):e01242-25. doi: 10.1128/mbio.01242-25 (PMC12345231; doi:10.1128/mbio.01242-25)
Supplement: Figure S1 — GRA1 is critical for rapid replication of Toxoplasma tachyzoites. [file mbio.01242-25-s0001.pdf]

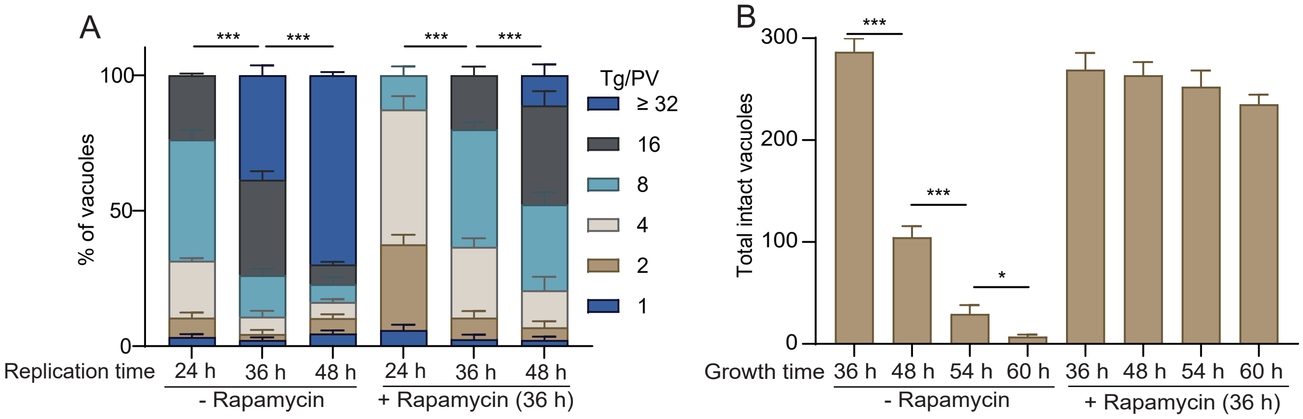

Fig S1. GRA1 is critical for rapid replication of *Toxoplasma* tachyzoites. (A) Intracellular replication assays of the iGRA1 parasites treated with rapamycin. The iGRA1 parasites were first treated with or without 50 nM rapamycin for 36 hours. Treated parasites were then allowed to infect fresh HFF cells and replicate for 24, 36 or 48 hours (without rapamycin). Subsequently the samples were fixed and the number of parasites in each parasitophorous vacuole (PV) was determined by IFA. Means ± SEM of three independent experiments, each with three replicates. *****P* < 0.0001, two-way ANOVA followed by Tukey's multiple comparison tests. (B) Natural egress of iGRA1 parasites treated with or without rapamycin. The iGRA1 parasites were first treated with or without 50 nM rapamycin for 36 hours and then used to infect fresh HFF monolayers. The parasites were cultured for additional 36, 48, 54 or 60 hours (without rapamycin) and the natural egress (rupture of PVs) was monitored by microscopy. In each experiment, the number of intact vacuoles from 30 random fields were counted. Mean ± SEM of three independent experiments for each condition, ****P* < 0.001, **P* < 0.05, student's t-test.
