# Supplementary material for: The critical role of Toxoplasma gondii GRA1 in nutrient salvage
Source: mBio. 2025 Jun 27;16(8):e01242-25. doi: 10.1128/mbio.01242-25 (PMC12345231; doi:10.1128/mbio.01242-25)
Supplement: Figure S2 — Metabolic changes in the iGRA1 strain after 36 hours of rapamycin treatment. [file mbio.01242-25-s0002.pdf]

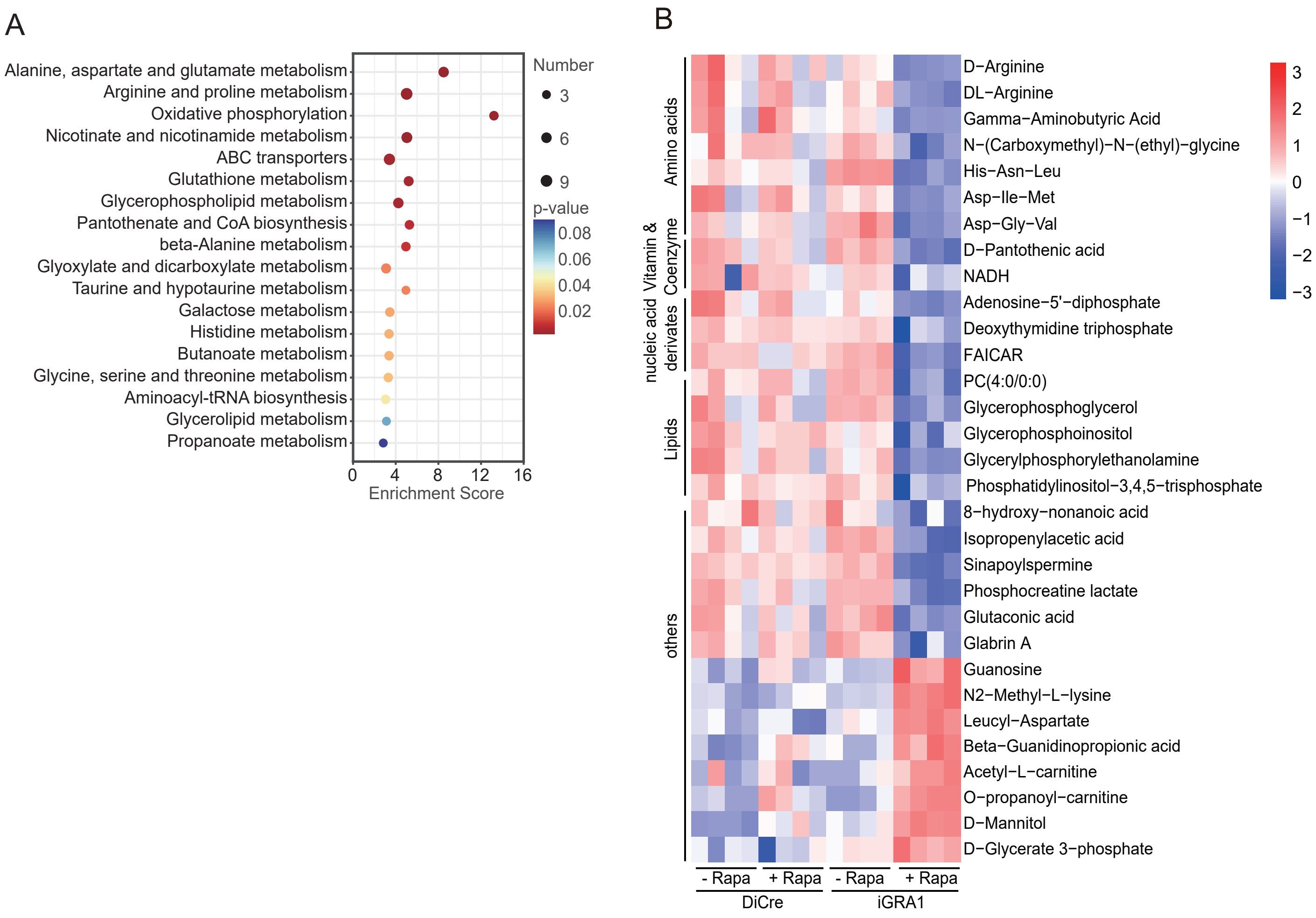

Fig S2. Metabolic changes in the iGRA1 strain after 36 hours of rapamycin treatment. The experiment was done in the same way as in Figure 3 C and D, except that the parasites were treated with (+ Rapa) or without (- Rapa) rapamycin for 36 hours instead of 44 hours before metabolic analyses.
