# Supplementary material for: The critical role of Toxoplasma gondii GRA1 in nutrient salvage
Source: mBio. 2025 Jun 27;16(8):e01242-25. doi: 10.1128/mbio.01242-25 (PMC12345231; doi:10.1128/mbio.01242-25)
Supplement: Figure S3 — Protein biotinylation in iGRA1 parasites determined by IFA. [file mbio.01242-25-s0003.pdf]

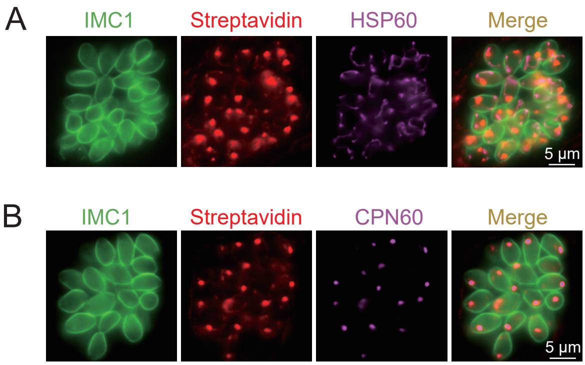

Fig S3. Protein biotinylation in iGRA1 parasites determined by IFA. Biotinylated proteins were detected by Alexa Fluor 594-conjugated streptavidin. Co-localization of biotinylated proteins with organelle specific markers was performed by co-staining with the mitochondrion marker HSP60 (A) or the apicoplast marker CPN60 (B).
