# Supplementary material for: The critical role of Toxoplasma gondii GRA1 in nutrient salvage
Source: mBio. 2025 Jun 27;16(8):e01242-25. doi: 10.1128/mbio.01242-25 (PMC12345231; doi:10.1128/mbio.01242-25)
Supplement: Figure S4 — Secretion of microneme proteins and dense granule proteins by extracellular parasites of the iGRA1 strain treated with or without rapamycin for 36 hours. [file mbio.01242-25-s0004.pdf]

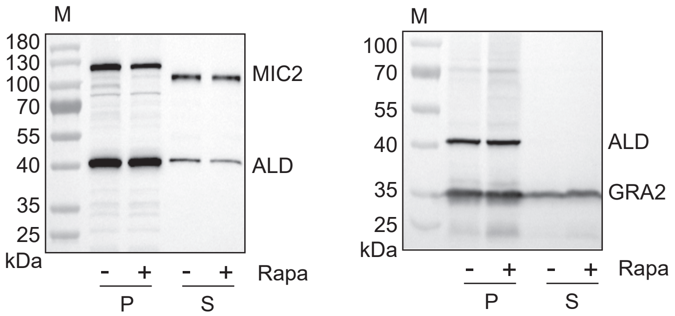

Fig S4. Secretion of microneme proteins and dense granule proteins by extracellular parasites of the iGRA1 strain treated with or without rapamycin for 36 hours. Secretion was induced by adding parasites into medium containing 3% BAS and 1% ethonal. GRA2, ALD and MIC2 in the supernatant (S) and pellet (P) fractions were analyzed by western blotting.
