# Supplementary material for: The critical role of Toxoplasma gondii GRA1 in nutrient salvage
Source: mBio. 2025 Jun 27;16(8):e01242-25. doi: 10.1128/mbio.01242-25 (PMC12345231; doi:10.1128/mbio.01242-25)
Supplement: Figure S5 — Alteration of MAG1 localization after GRA1 depletion under alkaline growth conditions. [file mbio.01242-25-s0005.pdf]

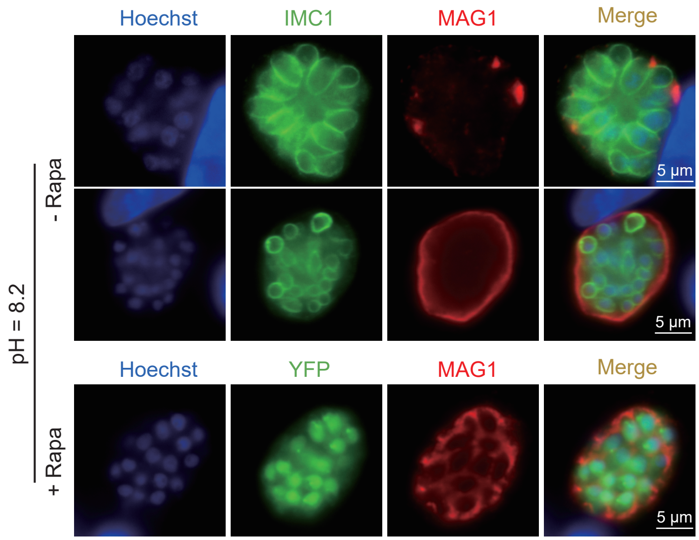

Fig S5. Alteration of MAG1 localization after GRA1 depletion under alkaline growth conditions. Parasites of the iGRA1/MAG1-3HA strain were first cultured in normal tachyzoite growth medium with (+ Rapa) or without (- Rapa) 50 nM rapamycin for 36 hours. Then, the parasites were collected and used to infect fresh HFF monolayers, and cultured for additional 72 hours under alkaline (pH = 8.2, ambient CO_2) growth conditions (without rapamycin). Subsequently the samples were fixed and examined by IFA, using an HA antibody to probe the localization of MAG1.
