# Supplementary material for: The critical role of Toxoplasma gondii GRA1 in nutrient salvage
Source: mBio. 2025 Jun 27;16(8):e01242-25. doi: 10.1128/mbio.01242-25 (PMC12345231; doi:10.1128/mbio.01242-25)
Supplement: Figure S6 — GRA1 is important for the growth of the type II strain ME49. [file mbio.01242-25-s0006.pdf]

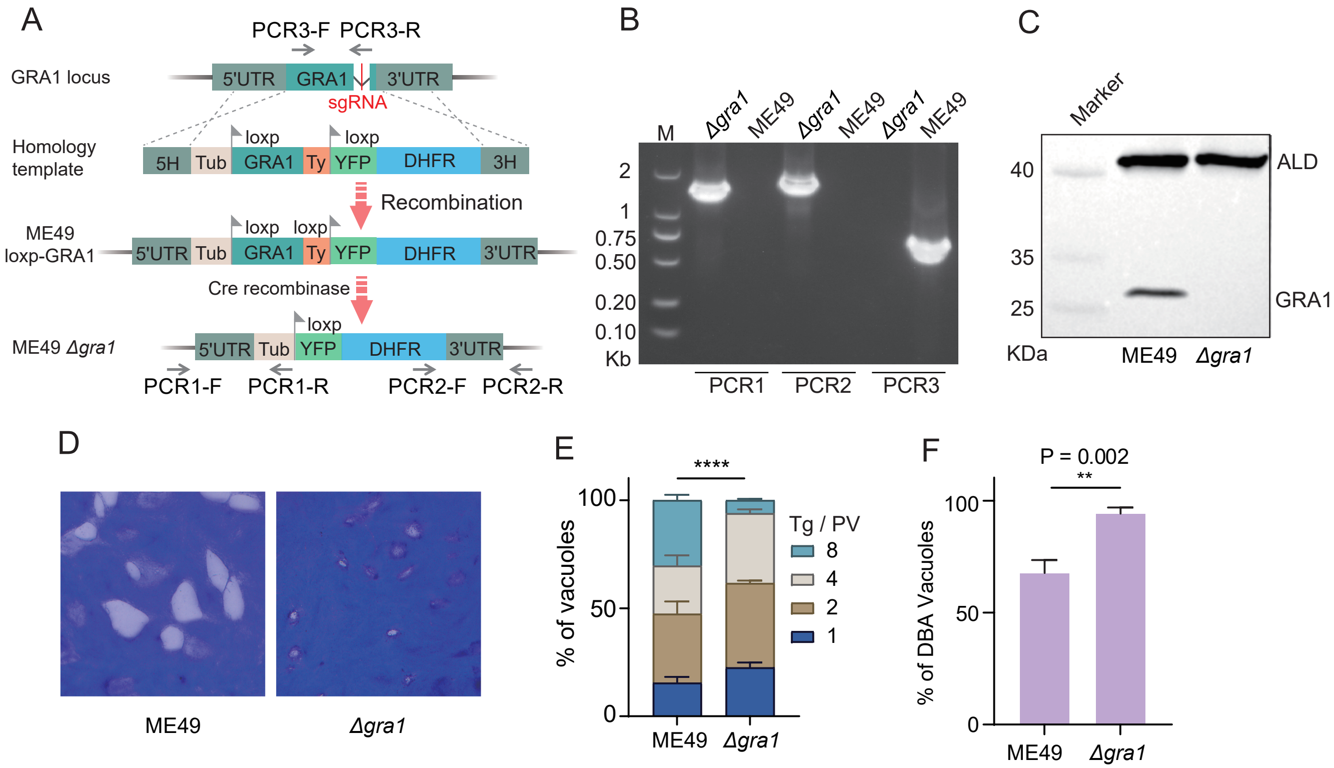

Fig S6. GRA1 is important for the growth of the type II strain ME49. (A) The strategy used to generate the GRA1 knockout strain ME49 *Δgra1*. The endogenous GRA1 locus was first floxed to construct the intermediate strain loxP-GRA1. Then, a plasmid expressing Cre recombinase was used to induce the excision of floxed GRA1 to obtain ME49 *Δgra1*, which turned YFP$^+$ after GRA1 deletion. (B) Diagnostic PCRs on a ME49 *Δgra1* clone. (C) Western blotting on a ME49 *Δgra1* clone to confirm the absence of GRA1 expression. ALD was included as a loading control. (D) Plaque assay comparing the overall growth of ME49 and ME49 *Δgra1*. (E) Intracellular replication assays of ME49 and ME49 *Δgra1*. Means ± SEM of three independent experiments, each with three replicates. *****P* < 0.0001, two-way ANOVA followed by Tukey's multiple comparison tests. (F) The percentage of DBA-positive vacuoles in strains cultured under alkaline conditions (pH 8.2) to induce bradyzoite formation. Means ± SD of three independent experiments, unpaired two-tailed Student's t-test.
