# Supplementary material for: The critical role of Toxoplasma gondii GRA1 in nutrient salvage
Source: mBio. 2025 Jun 27;16(8):e01242-25. doi: 10.1128/mbio.01242-25 (PMC12345231; doi:10.1128/mbio.01242-25)
Supplement: Table S1 — Plasmids used in this study. [file mbio.01242-25-s0007.docx]

Table S1 Plasmids used in this study

| **Name of plasmids** | **Use** | **Construction methods** |
| --- | --- | --- |
| pSAG1-Cas9-U6: sgUPRT | Template for gene specific CRISPR plasmid construction | Reference 1 |
| pSAG1-Cas9-U6: sgGRA1 | GRA1 specific CRISPR plasmid for the  construction of iGRA1 strain | Site-directed mutagenesis to replace the gRNA in pSAG1-Cas9-sgUPRT with gene specific gRNAs. |
| pSAG1-Cas9-U6: sgGRA2 | GRA2 specific CRISPR plasmid for the  construction of iGRA1-GRA2-3HA strain |  |
| pSAG1-Cas9-U6: sgGRA9 | GRA9 specific CRISPR plasmid for the  construction of iGRA1-GRA9-3HA strain |  |
| pSAG1-Cas9-U6: sgGRA14 | GRA14 specific CRISPR plasmid for the  construction of iGRA1-GRA14-3HA strain |  |
| pSAG1-Cas9-U6: sgGRA16 | GRA16 specific CRISPR plasmid for the  construction of iGRA1-GRA16-3HA strain |  |
| pSAG1-Cas9-U6: sgGRA17 | GRA17 specific CRISPR plasmid for the  construction of iGRA1-GRA17-3HA strain |  |
| pSAG1-Cas9-U6: sgMAG1 | MAG1 specific CRISPR plasmid for the  construction of iGRA1-MAG1-3HA strain |  |
| pDiCre-iGRA1 | Expression of loxp-GRA1-Ty-loxp-YFP-HXGPRT driven by a tubulin promoter | Homology template for the construction of DiCre-iGRA1 strain |
| ploxp-GRA1-ME49 | Expression of loxp-GRA1-Ty-loxp-YFP-DHFR driven by a tubulin promoter | Homology template for the construction of ME49-loxp-GRA1 strain |
| pUC19 | Template for pUC19 amplification | From the Sibley Lab |
| pSL24m-Linker-smFP-DHFR-LoxP-T7 | Homology template for the construction of GOI-3HA strains | Template plasmid containing smHA tag and DHFR drug selection marker |

**References:**

1. Shen B, Brown KM, Lee TD, Sibley LD. Efficient gene disruption in diverse strains of Toxoplasma gondii using CRISPR/CAS9. mBio 5, e01114-01114 (2014).
2. Xia N, Ye S, Liang X, Chen P, Zhou Y, Fang R, Zhao J, Gupta N, Yang S, Yuan J, Shen B. Pyruvate Homeostasis as a Determinant of Parasite Growth and Metabolic Plasticity in Toxoplasma gondii. mBio. 10(3):e00898-19(2019).
