# Supplementary material for: The critical role of Toxoplasma gondii GRA1 in nutrient salvage
Source: mBio. 2025 Jun 27;16(8):e01242-25. doi: 10.1128/mbio.01242-25 (PMC12345231; doi:10.1128/mbio.01242-25)
Supplement: Table S2 — Primers used in this study. [file mbio.01242-25-s0008.docx]

S2 Table. Primers used in this study

| **Primer** | **Sequence （5**`**– 3**`**）** | **Use** |
| --- | --- | --- |
| gRNA-GRA1-F | ATTCTATGCGTCGTCTGGAGGTTTTAGAGCTAGAAATAGC | To construct the GRA1 specific CRISPR plasmid |
| gRNA-GRA1-R | CTCCAGACGACGCATAGAATAACTTGACATCCCCATTTAC |  |
| GRA1-5H-F | GACCATGATTACGCCGATATACACAGTGCCAGTCG | Amplification of 5`-homology of GRA1 for pDiCre-iGRA1 construction |
| GRA1-5H-R | TAAGACTGGCCGTCGCTTGCTTGATTTCTTCAAAGAACA |  |
| GRA1-3H-F | TTAATTGCGCGCTTGTTGGAGGTAGCTTTGTTGTG | Amplification of 3`-homology of GRA1 for pDiCre-iGRA1 construction |
| GRA1-3H-R | GCCAGTGAATTCGAGGTTGACACAATCCGACGAGT |  |
| GRA1-CDS-F | TAGAATTCCGACAAAATGGTGCGTGTGAGCGCT | Amplification of CDS for pDiCre-iGRA1 construction |
| GRA1-CDS-R | GTTCGTGTGGACCTCCTCTCTCTCTCCTGTTAGGA |  |
| PUC19-F | GAGGTCCACACGAACCAGGA | Amplification of pUC19-YFP-HXGPRT for pDiCre-iGRA1 |
| PUC19-R | GGCGTAATCATGGTCATAGC |  |
| Tublin-F | CGACGGCCAGTCTTAAGC | Amplification of Tublin for pDiCre-iGRA1 construction |
| Tublin-R | TTTGTCGGAATTCTATAACTTCGTAT |  |
| GRA1-PCR1-F | ACCACTACAGGTCCTCCAAC | PCR1 of DiCre-iGRA1 strain |
| GRA1-PCR1-R | CGTTTGGTGGATGTCTTCTG |  |
| GRA1-PCR2-F | GCTACGACTTCAACGAGATG | PCR2 of DiCre-iGRA1 strain |
| GRA1-PCR2-R | GCCACGCTTACGTATTACAC |  |
| GRA1-PCR3-F | TGCGTGTGAGCGCTATTGTC | PCR3 of DiCre-iGRA1 strain |
| GRA1-PCR3-R | GCCACGCTTACGTATTACAC |  |
| gRNA-GRA2-F | GTGCGTGTATCAGTTGTGCGGTTTTAGAGCTAGAAATAGC | To construct the GRA2 specific CRISPR plasmid |
| gRNA-GRA2-R | CGCACAACTGATACACGCACAACTTGACATCCCCATTTAC |  |
| GRA2-3HA-F | TGGAGCCCCAACAGCGGGCCGCACACGTGCCCGTCCCAGACTTTTCGCAGGGCTACCCCTACGATGTG | Amplification of 3HA-DHFR for GRA2-3HA strain |
| GRA2-3HA-R | GTCACATACAACCAACTGACGTCCGGCATTGGGCTATTGCGTACATACCACACCGCTTTCTCAACAGG |  |
| gRNA-GRA9-F | GCGGAACACTCTGCCAGAAAGTTTTAGAGCTAGAAATAGC | To construct the GRA9 specific CRISPR plasmid |
| gRNA-GRA9-R | TTTCTGGCAGAGTGTTCCGCAACTTGACATCCCCATTTAC |  |
| GRA9-3HA-F | CTGTGACGTCCGTCGAACTGCCCCGACTCTCGCAGGAAGACCGAGGACTCGGCTACCCCTACGATGTG | Amplification of 3HA-DHFR for GRA9-3HA strain |
| GRA9-3HA-R | GAGAATGCTACGAACGCAAAATTGAGTGCGTCCTAAACGCTGCGCACACGCACCGCTTTCTCAACAGG |  |
| gRNA-GRA16-F | GTGTTCGTGCAGATTAAGCGGTTTTAGAGCTAGAAATAGC | To construct the GRA16 specific CRISPR plasmid |
| gRNA-GRA16-R | CGCTTAATCTGCACGAACACAACTTGACATCCCCATTTAC |  |
| GRA16-3HA-F | ACGAAGATGATTTTGACTGTTCACGCGCGAAGCGGAAAAATGATCAGATGGGCTACCCCTACGATGTG | Amplification of 3HA-DHFR for GRA16-3HA strain |
| GRA16-3HA-R | CGGTCAACCAAAGTTGCAAGAAGAAACAACATGCACCACATCTACTGTCACACCGCTTTCTCAACAGG |  |
| gRNA-GRA17-F | ACACTGGCACTTCTTCCACCGTTTTAGAGCTAGAAATAGC | To construct the GRA17 specific CRISPR plasmid |
| gRNA-GRA17-R | GGTGGAAGAAGTGCCAGTGTAACTTGACATCCCCATTTAC |  |
| GRA17-3HA-F | CCCTGAGTGCGAAGATGGCCGTGAAGCAGAAGGCCATGCAGGGCAAGCAGGGCTACCCCTACGATGTG | Amplification of 3HA-DHFR for GRA17-3HA strain |
| GRA17-3HA-R | AGTCTCTCACCAGCTCCAACCGAAGTCCCTCTCCACAGACTCTTTCATACCACCGCTTTCTCAACAGG |  |
| gRNA-MAG1-F | AGCAGAATGATCGTGTATCGGTTTTAGAGCTAGAAATAGC | To construct the MAG1 specific CRISPR plasmid |
| gRNA-MAG1-R | CGATACACGATCATTCTGCTAACTTGACATCCCCATTTAC |  |
| MAG1-3HA-F | CGTCTAAAGAAGCGGAGGAAGCTCGTCAGATCTTAGCGGAACAGGCAGCTGGCTACCCCTACGATGTG | Amplification of 3HA-DHFR for MAG1-3HA strain |
| MAG1-3HA-R | CGAAGGGCAGAAACCGGCGCTGTTGCATAGACACTGCGCCTTGAACCCAGCACCGCTTTCTCAACAGG |  |
